# Efficacy and safety of pharmacotherapy for Alzheimer’s disease and for behavioural and psychological symptoms of dementia in older patients with moderate and severe functional impairments: a systematic review of controlled trials

**DOI:** 10.1186/s13195-021-00867-8

**Published:** 2021-07-16

**Authors:** M. Seibert, V. Mühlbauer, J. Holbrook, S. Voigt-Radloff, S. Brefka, D. Dallmeier, M. Denkinger, C. Schönfeldt-Lecuona, S. Klöppel, C. A. F. von Arnim

**Affiliations:** 1 Department of Neurology, University Clinic Ulm, Ulm, Germany; 2 Agaplesion Bethesda Clinic, Institute for Geriatric Research, Ulm University, Ulm, Germany; 3 Geriatric Center Ulm/Alb-Donau, Ulm, Germany; 4 Center for Geriatric Medicine and Gerontology, Medical Center and Faculty of Medicine, University of Freiburg, Freiburg im Breisgau, Germany; 5 Institute for Evidence in Medicine (for Cochrane Germany Foundation), Medical Center and Faculty of Medicine, University of Freiburg, Freiburg im Breisgau, Germany; 6 Department of Epidemiology, Boston University School of Public Health, Boston, USA; 7 Department of Psychiatry and Psychotherapy III, University Clinic Ulm, Ulm, Germany; 8 University Hospital of Old Age Psychiatry, University of Bern, Bern, Switzerland; 9 Division of Geriatrics, University Medical Centre, Georg August University, Robert-Koch-Str. 40, 37075 Göttingen, Germany

**Keywords:** Frail elderly, Alzheimer’s disease, Dementia, BPSD, Systematic review, Drug therapy

## Abstract

**Background:**

Many patients with Alzheimer’s disease (AD) are physically frail or have substantial functional impairments. There is growing evidence that such patients are at higher risk for medication-induced adverse events. Furthermore, frailty seems to be more predictive of poor clinical outcomes than chronological age alone. To our knowledge, no systematic review of clinical trials examining drug therapy of AD or behavioural and psychological symptoms of dementia (BPSD) has specifically focused on the topic of physical frailty. Our objective was to evaluate the efficacy and safety of pharmacotherapy in AD patients with frailty or significant functional impairments.

**Methods:**

We performed a systematic literature search in MEDLINE, Embase and the Cochrane Central Register of Controlled Trials (CENTRAL) for randomized controlled trials (RCTs) of drug therapy of AD and BPSD in patients with significant functional impairments according to the Preferred Reporting Items for Systematic Reviews and Meta-Analyses (PRISMA) statement and Cochrane research criteria. Significant functionally impaired patient populations were identified using the recommendations of the Medication and Quality of Life in frail older persons (MedQoL) Research Group. Screening, selection of studies, data extraction and risk of bias assessment were performed independently by two reviewers. Outcomes including functional status, cognitive function, changes in BPSD symptoms, clinical global impression and quality of life were analysed. For assessing harm, we assessed adverse events, drop-outs as a proxy for treatment tolerability and death. Results were analysed according to Cochrane standards and the Grading of Recommendations Assessment, Development and Evaluation (GRADE) approach.

**Results:**

Of 45,045 search results, 38,447 abstracts and 187 full texts were screened, and finally, 10 RCTs were included in the systematic review. Selected articles evaluated pharmacotherapy with acetylcholinesterase-inhibitors (AChEI), anticonvulsants, antidepressants and antipsychotics. Studies of AChEIs suggested that patients with significant functional impairments had slight but significant improvements in cognition and that AChEIs were generally well tolerated. Studies of antidepressants did not show significant improvements in depressive symptoms. Antipsychotics and anticonvulsants showed small effects on some BPSD items but also higher rates of adverse events. However, due to the very small number of identified trials, the quality of evidence for all outcomes was low to very low.

Overall, the small number of eligible studies demonstrates that significantly functional impaired older patients have not been adequately taken into consideration in most clinical trials investigating drug therapy of AD and BPSD.

**Conclusion:**

Due to lack of evidence, it is not possible to give specific recommendations for drug therapy of AD and BSPD in frail older patients or older patients with significant functional impairments. Therefore, clinical trials focussing on frail older adults are urgently required. A standardized approach to physical frailty in future clinical studies is highly desirable.

**Supplementary Information:**

The online version contains supplementary material available at 10.1186/s13195-021-00867-8.

## Introduction

People over the age of 75 years are the world’s fastest-growing demographic group. Health systems worldwide must meet new challenges generated by the ageing population, including the medical care of frail older patients [1, 2]. With increasing population age, the prevalence of age-associated conditions such as Alzheimer’s disease (AD) and frailty will also rise [3, 4]. Factors such as frailty or disability appear to be more predictive for detrimental outcomes (e.g., mortality) than chronological age alone [5–7]. Although the International Council for Harmonisation of Technical Requirements for Pharmaceuticals for Human Use (ICH) recommended the inclusion of frail older patients in RCTs as far back as 1993, these patients are still seldom included in RCTs [8–11]. Therefore, although such patients are overrepresented in real-world clinical settings, as they often have multiple morbidities and are recipients of polypharmacy, they are underrepresented in the studies that provide the data on which clinical treatments are based [12]. Due to the lack of available evidence, frailty is also often ignored during the formulation of medical guidelines [13]. It is well-known that medications may have different effects in this vulnerable population: impaired functional status, systemic illness and metabolic changes may result in different pharmacologic and pharmacodynamic responses, a different adverse event profile and ultimately a different risk-benefit calculus [12, 14, 15].

Despite its importance as a medical concept, there is still no clear universally accepted definition of frailty. Frailty is generally described as an age-associated syndrome with increased vulnerability to minor stressor events because of impairments in multiple systems [16, 17]. The concept of frailty takes biological age into account rather than chronological age. Many different approaches are used to estimate frailty [18, 19]. The two most used are the (i) Frailty Phenotype by Fried et al. and the (ii) Frailty Index (FI) by Rockwood and Mitnitski. The so-called Frailty Phenotype mainly applies to physical frailty, as assessed by reduced grip strength, unintended weight loss, exhaustion, decreased physical activity and slow gait speed [16]. Rockwood and Mitnitski have proposed defining frailty by an accumulation of deficits, including but not limited to functional items, and mainly relying on comorbidities, including cognitive impairments [20]. Frailty is however in both concepts tightly linked to functional status. Patients with AD have more comorbidities and are more often physically frail than patients without AD [21–23]. Conversely, some evidence indicates that frail older patients may have a higher risk of developing AD; furthermore, higher frailty scores are associated with a more rapid cognitive decline [24, 25]. Frailty may also result in a higher risk of adverse events from drug therapy for AD, for instance, during the use of antidementia medications such as acetylcholinesterase inhibitors [14, 26].

Frailty of AD patients also correlates with the severity of behavioural and psychological symptoms of dementia (BPSD, e.g. psychosis, depression, apathy, agitation, aggression and sleep disturbances) and caregiver burden [27, 28]. Reciprocally, AD patients with BPSD are more likely to be frail and may also have a higher risk for adverse events such as falls during drug therapy [29]. Drug therapy of BPSD in patients with AD is usually recommended only when psychosocial interventions or other non-pharmacological interventions are not sufficient [30]. BPSD is a substantial consideration in patients with AD and has a major impact on patients’ and caregivers’ quality of life [31, 32]. Common therapies for BPSD often consist of psychopharmacotherapy (e.g. antipsychotics, antidepressants or even anticonvulsants), many of which may have anticholinergic properties and are possibly inappropriate for geriatric patients, as indicated in the Beers’ criteria list or the PRISCUS list [33, 34].

Despite the broad importance of drug therapy for the treatment of cognitive and BPSD symptoms in frail AD patients, to our knowledge, there has been no systematic review in this population, even though the available literature suggests a different risk-benefit ratio due to a higher rate of adverse events. Current evidence mainly relies on a mere handful of RCTs. The objective of this review was to determine the efficacy and safety of drug therapy for frail older patients with Alzheimer’s disease and associated BPSD. Is there sufficient evidence to recommend safe and effective treatments for this vulnerable patient population?

## Methods

The review process was performed according to the Preferred Reporting Items for Systematic Reviews and Meta-Analyses (PRISMA) statement and the Cochrane Handbook for Systematic Reviews of Interventions [35, 36].

### Types of studies

We included double-blind RCTs comparing pharmacotherapy of AD or BPSD in AD with placebo or other drug interventions and involving frail older patients. Studies in any settings were included (outpatient, inpatient, long term care facilities or nursing homes). Studies including patients with dementia or with neuropsychiatric symptoms not in the context of AD were excluded.

### Types of participants

The patients in included trials had to be diagnosed with AD according to internationally accepted criteria such as the Diagnostic and Statistical Manual of Mental Disorders criteria IV and 5 (DSM IV/DSM-5) [37, 38], International Classification of Diseases 10 (ICD-10) [39] or the National Institute of Neurological and Communicative Disorders and Stroke-Alzheimer’s Disease and Related Disorders Association (NINCDS-ADRDA) criteria [40]. Studies were also eligible if AD patients constituted a major part of the study population (> 50%), even if other types of dementia were also included. Patient populations with BPSD resulting from AD were also considered. A mean age of 70 years or more or a minimum age of 65 years was required.

### Physical frailty/functional impairment evaluation

To evaluate physical frailty and/or significant functional impairments, the patient population in RCTs was classified according to the *Medication and Quality of Life in Frail Older Persons (MedQoL) Research Group* criteria, which define cut-offs for 51 established scores and differentiates between functionally independent, functionally slightly impaired, functionally significantly impaired/partially dependent and functionally severely impaired/disabled/mostly or totally dependent [41]. The study population had to be rated on average as at least “significantly impaired or partially dependent” to allow inclusion in this review. However, studies in which frailty was defined mainly based on cognitive impairment were excluded, because this could have resulted in AD patients being included only on the basis of their associated cognitive deficits. This was discussed within the MedQoL Research Group and mutually agreed upon. Using this methodology, we identified study patient populations that were likely to be physically frail or significantly functionally impaired (but not primarily due to cognitive deficits).

### Types of interventions

Any pharmacotherapies for AD and BPSD in any dosage or treatment duration were included.

### Types of outcome measures

The following outcomes were defined [42]:

- Functional status as rated by MedQoL criteria [41],
- Cognitive function (as measured by psychometric tests),
- Changes in BPSD symptoms (as measured by psychometric tests or questionnaires),
- Clinical global impression, and
- Quality of life.

For assessing harm, we determined the outcomes:

- Adverse events,
- Drop-outs as a proxy for treatment tolerability, and
- Death.

These outcomes correspond to the AD-recommended outcomes for AD trials by the IQWiG (Institute for Quality and Efficiency in Health Care) and the EMA (European Medicines Agency) [43, 44].

### Search methods for identification of studies

We searched the following databases: Embase, MEDLINE and the Cochrane Central Register of Controlled Trials (CENTRAL), on 24/06/2017. There was no restriction on publication language. An update search was performed on 15/01/2019. We defined 1 January 1992 as the publication period’s lower limit, as it was about the time of the introduction of the term “frailty” in current literature [45].

In addition, the German national guidelines for therapy of AD and BPSD and the references from systematic reviews were also screened for relevant studies. All identified publications were imported in Covidence^1^ [46] and then independently screened by two reviewers (VM, MS). If the authors disagreed on a study’s inclusion, discussion was continued until consensus was reached; otherwise, a third review author (CvA) was consulted.

### Data extraction and risk of bias assessment

Data were independently extracted by two review authors (VM, MS) using a standardized data collection form by the Cochrane Effective Practice and Organisation of Care (EPOC) group [47]. Whenever data were not reported or were not suitable for extraction the corresponding author was contacted. Missing standard deviations (SD) were calculated using other reported statistical data.

The risk of bias assessment was performed independently by two review authors (VM, MS) using the Cochrane Collaboration’s tool for assessing risk of bias in RCTs [48].

For each publication, the risk of bias was rated as high, low or unclear. If authors disagreed, discussion was continued until consensus was reached; otherwise, a third review author (CvA) was consulted.

### Data analysis

We calculated mean differences (MD) or standardized mean differences (SMD) for continuous outcomes or the risk ratio (RR) for dichotomous outcomes, and the corresponding 95% confidence interval (CIs) using a fixed-effect model. A probability value of < 0.05 was determined to be the significance level.

Heterogeneity was assessed using the I^2^ test. For probably relevant heterogeneity (I^2^ value ≥ 50%), possible causes were examined, and a random-effect model was used.

We analysed shorter ordinal scores such as the Clinical Global Impression - Improvement Scale (CGI-I, a 7-point Likert scale) as dichotomous outcomes by combining adjacent categories into two groups: “worsening/no change” or “clinical improvement”.

When analysing outcomes of cross-over trials, potentially relevant carry-over effects or other issues regarding the cross-over study design were handled by only including study data of the first treatment period.

The statistical analysis was performed using Review Manager 5.3 (RevMan) by the Cochrane Collaboration (MS) [49].

### Quality of evidence ratings

Quality of evidence was assessed using the Grading of Recommendations Assessment, Development and Evaluation (GRADE) approach [50, 51]. GRADE ratings were performed by two review authors independently (VM, MS) according to recommendations of the GRADE handbook [52].

Using the GRADE approach, quality of evidence (very low/low/moderate/high) for most important outcomes are rated separately using the following explicit criteria: study design, risk of bias, imprecision, inconsistency, indirectness and magnitude of effect. The following outcome groups were considered as most important and included in GRADE ratings: functional status, cognitive function, BPSD, adverse events, treatment tolerability, death and quality of life.

Imprecision ratings for continuous outcomes were performed considering the minimally important difference (MID) (see Additional file 1).

If there was no published MID, Hedges’ g was calculated to determine potentially relevant treatment effects. An effect size (Hedges’ g) of ≥0.2 was considered as an MID, whereas Hedges’ g < 0.2 was considered as no relevant treatment effect. For further classification of the magnitude of the effect size we used the recommendations by Cohen: 0.2 = small, 0.5 = medium, 0.8 = large [53]. A study population of fewer than 400 was considered as too small to ensure adequate precision of outcomes [54].

When rating imprecision of outcomes for dichotomous data, the figure by Guyatt et al. with a 25% relative risk reduction (RRR) was used to estimate the acceptable size for a study population. In addition, a 95% CI including a RR of at least 0.75 to 1.25 was considered as large [54]. GRADE ratings and the creation of summary of findings tables were performed using GRADEpro [55].

## Results

**Fig. 1:**
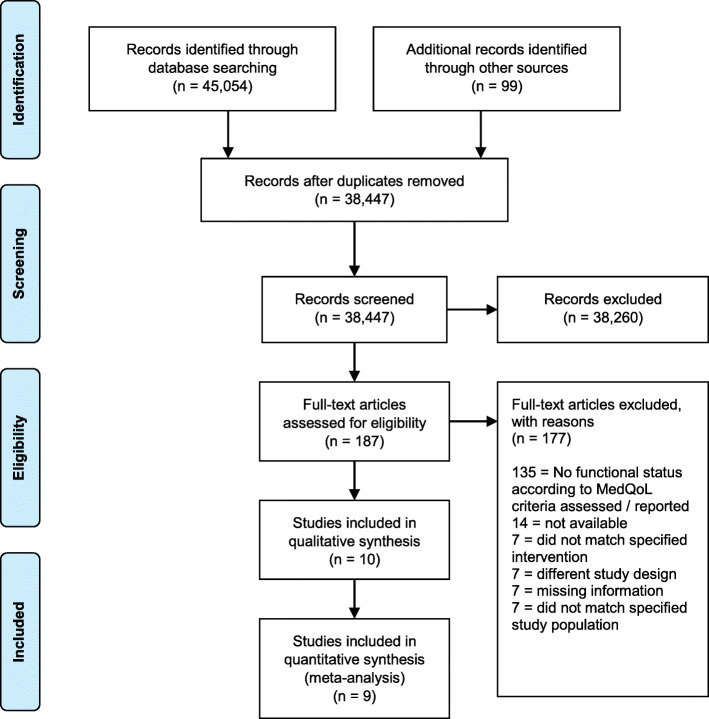
Flowchart of the selection strategy of studies according to PRISMA protocol

The online searches performed in 2017 and 2019 retrieved 45,045 records. In addition, the search for potentially relevant publications used in systematic reviews and of the German national guidelines for dementia retrieved 99 records. After removing duplicates, 38,447 titles and abstracts were screened, and 187 full texts were read. Reasons for exclusion are shown in Fig. 1. Finally, 10 studies were included in this systematic review.

### Characteristics of included studies

All study populations were at least 70 years on average and mostly “significantly impaired” according to MedQoL criteria. Included studies used the PSMS, the FIM or the MDS-ADL scales, which we used to assess impairment in physical function for frailty evaluation according to MedQoL criteria (see Additional file 2). Ten RCTs were included in the systematic review: two studies evaluated AChEIs, four studies anticonvulsants, two studies antidepressants, one study antipsychotics and one triple-arm study investigated one antidepressant and one antipsychotic medication. Female patients constituted the majority of participants (61–100%) in every treatment group in the included studies, except for the fluoxetine group of Petracca et al. (2001) (47% female) [56]. All studies used the oral drug administration route only. Tolerability and safety were assessed by the number and types of adverse events and the drop-out rate during the treatment period. Quality of life was not assessed in any of the studies. For more detailed study information, see Table 1.

**Table 1:** Characteristics of included studies

	Study (Author, year)	Duration	Sample size (n)	Age of study population (IG/CG)	Baseline MMSE (IG/CG)	Intervention	Endpoints	Results
**AChEI**	Burns et al., 2009 [57]	6 months	407	83.7/83.5	8.8/9.1	Galantamine: 24 mg/day target dose (12 mg twice a day). Dose reduction to 8 mg twice a day to improve tolerability was possible. Placebo.	MDS-ADL, SIB	Significantly improved cognitive function. No significant improvement in the co-primary outcome of ADLs.
	Tariot et al., 2001 [58]	24 weeks	208	85.4/85.9	14.4	Donepezil: 10 mg/day target dose. Dose reduction to improve tolerability was possible. Placebo.	CDR-SoB, MMSE, NPI-NH, PSMS	Donepezil-treated patients improved or maintained in cognition. Impact of donepezil on BPSD remains unclear.
**Antidepressants**	Petracca et al., 1996 [59] (cross-over trial)	2 × 6 week treatment period, separated by a 2-week wash-out period	24	71.5/72.4	21.0/22.1	Clomipramine: 100 mg/day target dose. Placebo.	FIM, HAM-D, MMSE	Clomipramine was significantly more effective in lowering depression scores compared to placebo. No changes in ADL measures.
	Petracca et al., 2001 [56]	6 weeks	41	70.2/71.3	23.2	Fluoxetine: 40 mg/day target dose. Placebo.	CGI-I, FIM, HAM-A, HAM-D, MMSE	No significant differences in treatment effects on depression comparing fluoxetine and placebo.
**Anticonvulsants**	Olin et al., 2001 [60]	6 weeks	21	74.7	5.9/6.1	Carbamazepine: 400 mg/day target dose. Placebo.	BPRS, CGI-I, HAM-D, IADL, MMSE, PSMS	Modest clinical benefit in global impression and a particular benefit for hostile behaviour in carbamazepine-treated patients was shown.
	Porsteinsson et al., 2001 [61]	6 weeks	56	85.3/84.7	7.0/6.7	Carbamazepine: 375 mg/day starting dose, followed by flexible dose regimen. Placebo.	BPRS, CERAD BRSD, CGI-I, MMSE, OAS, PSMS	Possible short-term efficacy of valproate in reduction of agitation in patients with dementia in the nursing home.
	Tariot et al., 1994 [62] (cross-over trial)	2 × 5 week treatment period, separated by a 2 week wash-out period	25	84.5	7.6	Carbamazepine: 100–800 mg/day based on physician’s review.	BPRS, CGI-I, DMAS, MMSE, OAS, PSMS	Short-term therapy with Carbamazepine may have beneficial effects on BPSD in patients with dementia and agitation (significant reduction in BPRS total score).
	Tariot et al., 1998 [63]	6 weeks	51	87.1/84.8	3.9/8.3	Carbamazepine: 100 mg/day starting dose, increased by 50 mg/day every 2–4 days; in the absence of toxicity a serum level of 5–8 μg/ml was maintained. Placebo.	BPRS, CERAD BRSD, CGI-I, MMSE, PSMS	Carbamazepine showed significant short-term efficacy for agitation. Significant reduction of the BPRS agitation and hostility factor compared with placebo.
**Antipsychotics**	Tariot et al., 2006 [64]	10 weeks	284	Q: 81.9 H: 83.6 P: 83.9	Q: 12.4 H: 12.7 P: 13.2	Quetiapine: 100 mg/day target dose, maximum dose of 600 mg/day according to clinical response and tolerability Haloperidol: 2 mg/day target dose, maximum dose of 12 mg/day according to clinical response and tolerability. Placebo.	AIMS, BPRS, CGI-S, MMSE, MOSES, NPI-NH, PSMS, SAS	No significant improvement in BPRS total scores. Inconsistent significant improvement in some parts of BPSD for haloperidol treated patients. Tolerability was better for quetiapine compared with haloperidol.
**Antipsychotics/antidepressants**	Teranishi et al., 2013 [65]	8 weeks	82	R: 80.7 F: 83.2 Y: 83.5	R: 5.2 F: 4.5 Y: 4.4	Flexible oral dosing regimen. Risperidone: 0.5–2 mg/day target dose. Fluvoxamine: 25–200 mg/day target dose. Yokukansan: 2.5–7.5 g/day target dose.	DIEPSS, FIM, MMSE, NPI-NH	NPI-NH scores decreased in all three groups with no significant differences. Tolerability for yokukansan and quetiapine seemed to be more favourable than for risperidone.

*CG* control group, *F* fluvoxamine, *H* haloperidol, *IG* intervention group, *P* placebo, *Q* quetiapine, *R* risperidone, *Y* yokukansan; Endpoints: *AIMS* Abnormal Involuntary Movement Scale, *BPRS* Brief Psychiatric Rating Scale, *CDR-SoB* Clinical Dementia Rating – Sum of Boxes, *CERAD BRSD* Behavior Rating Scale for Dementia of the Consortium to Establish a Registry for Alzheimer’s Disease, CGI-I, Clinical Global Impression of Improvement, *CGI-S* Clinical Global Impression of Illness Severity, *DIEPSS* Drug-Induced Extra-Pyramidal Symptom Scale, *DMAS* Dementia Mood Assessment Scale, *FIM* Functional Independence Measure, *HAM-A/-D* Hamilton Rating Scale for Anxiety/Depression, *IADL* instrumental activities of daily living by Lawton and Brody, MDS-ADL, Minimum Data Set – Activities of Daily Living, *MMSE* Mini-Mental State Examination, *MOSES* Multidimensional Observation Scale for Elderly Subjects, *NPI-NH* Neuropsychiatric Inventory – Nursing Home Version, *OAS* Overt Aggression Scale, *PSMS* Physical Self-Maintenance Scale, *SAS* Simpson-Angus Scale, *SIB* Severe Impairment Battery

#### Acetylcholinesterase inhibitors

Burns et al. compared galantamine with placebo in patients in residential homes, nursing homes or geriatric residences with severe AD in 57 European investigational sites using a multicentre design [57], and Tariot et al. focussed on patients in nursing homes with moderate AD in 27 US nursing homes and compared donepezil with placebo [58]. Both trials’ treatment duration was about 6 months.

#### Antidepressants

Two studies were included investigating the efficacy and safety of antidepressants (fluoxetine or clomipramine) in outpatients with depression and mild AD in Argentina for 6 weeks [56, 59]. Unfortunately, the cross-over trial by Petracca et al. (1996) reported no suitable data for extraction and no additional study data could be acquired [59]. Therefore, the study data were only analysed qualitatively, except for adverse events of the first treatment period.

#### Antidepressants/antipsychotics

We identified a 3-arm study comparing the efficacy and safety of flexibly-dosed fluvoxamine, risperidone and the traditional Japanese herbal medicine yokukansan for 8 weeks in patients with severe dementia and BPSD in a psychiatric hospital in Japan [65]. Yokukansan (TJ-54) is part of traditional Japanese herbal medicine and is used to treat insomnia, irritability and neurological disorders such as dementia and AD [66] and may have neuroprotective effects against glutamate-induced excitotoxicity [67]. Yokukansan was used as the control group in the further analysis of the study data. Teranishi et al. also included a small number of patients with vascular dementia (VD) or dementia with Lewy bodies (DLB) [65].

#### Antipsychotics

A multicentre, 3-arm RCT compared treatment with flexibly dosed haloperidol, quetiapine or placebo in patients in nursing homes with AD and psychotic symptoms in 47 investigational sites in the USA for 10 weeks [64].

#### Anticonvulsants

Four studies investigating the efficacy and safety of anticonvulsants for aggressive or agitated behaviour in patients with AD, vascular or mixed dementia were included. Three RCTs investigated treatment with carbamazepine [60, 62, 63], while Porsteinsson et al. assessed the use of valproate [61]. Subjects were living in US nursing homes, long-care facilities or with a caregiver.

### Excluded studies

As shown in Fig. 1, most studies were excluded due to missing functional status assessments or because no suitable assessment of physical frailty according to MedQoL criteria was available. Another main reason for exclusion was study populations’ average age, lack of a drug intervention or availability as an abstract only. In some of the screened full texts, potentially suitable physical frailty assessments according to MedQoL criteria were reported, but not measured at baseline.

### Risk of bias assessment

**Fig. 2:**
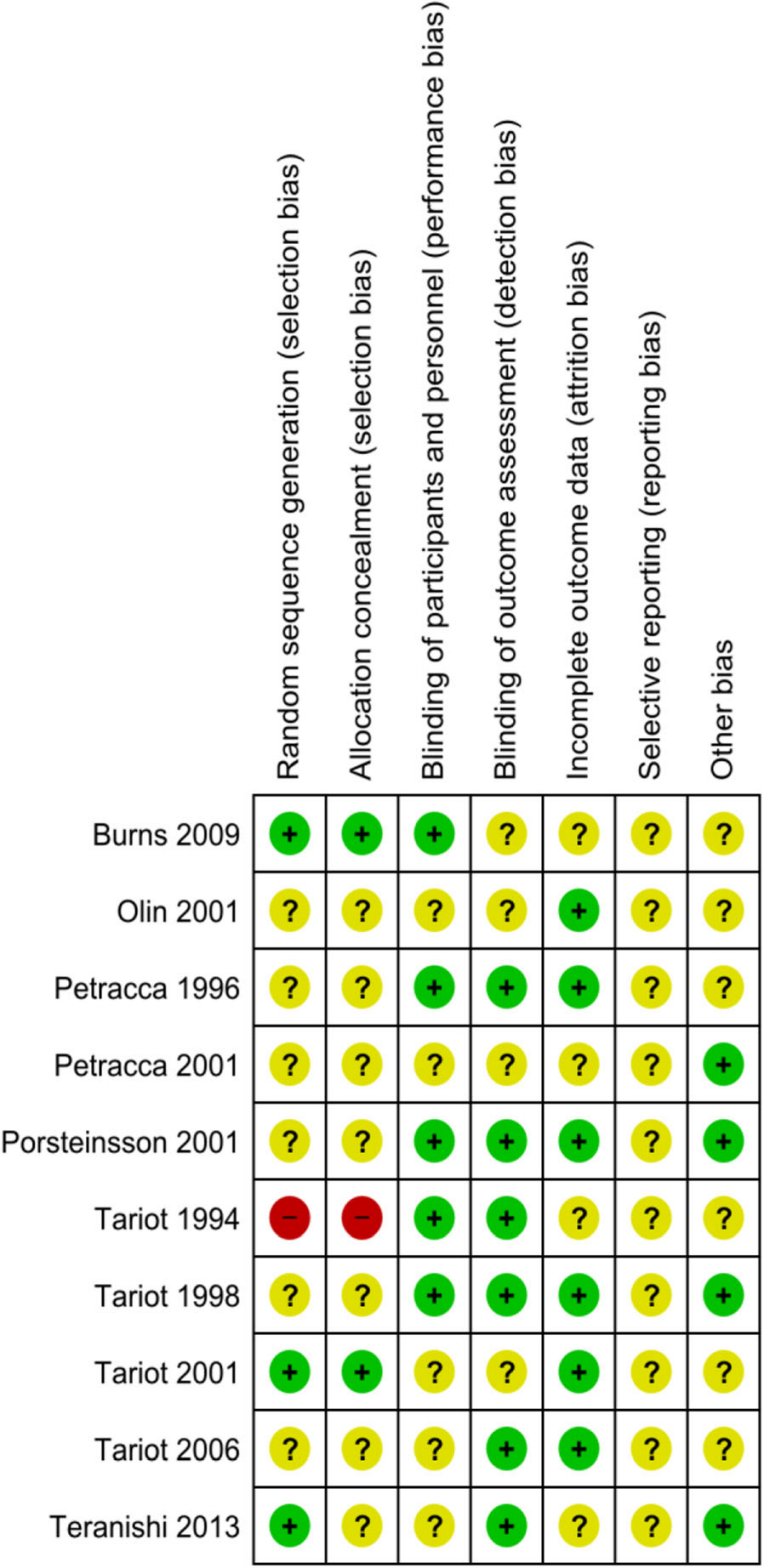
Risk of bias summary: review authors’ judgements about each risk of bias item for each included study

Overall, most studies were at unclear or low risk of bias in each of the assessed domains. The only trial with a probable high risk of bias (Tariot et al., 1994 [62]) was not included in the further quantitative analysis, due to high risk of selection bias and insufficient reporting of outcome data. Risk of bias assessment for each domain of the included RCTs is presented in Fig. 2.

### Acetylcholinesterase inhibitors

Two studies investigating the efficacy and safety of AChEI (galantamine, donepezil) were included [57, 58]. All outcomes rated according to GRADE are summarized in an evidence profile (see Additional file 3).

#### Functional status

Neither of the trials showed a significant change in functional status as assessed with the MDS-ADL (co-primary outcome in Burns et al.) and PSMS (secondary outcome in Tariot 2001 et al.) [57, 58]. Both intervention and control groups’ functional status worsened slightly during the study period. Unfortunately, only one study reported the assessment of physical frailty in a way suitable for data extraction [57]. Therefore, only Burns et al. was included in the rating of the quality of evidence for functional status involving 364 participants (MD − 0.40; 95% CI: [− 1.32, 0.52]) [57]. The quality of evidence of this outcome according to GRADE was very low.

#### Cognitive function

When assessing cognitive function, Burns et al. [57] reported a significant difference in cognitive function assessed as a co-primary outcome with the Severe Impairment Battery (SIB) in favour of galantamine over placebo (MD 5.2; 95% CI: [2.24, 8.16]; Hedges’ g = 0.36) [57]. Tariot et al. (2001) [58] did not provide suitable data for extraction, but reported superiority of donepezil compared with placebo as measured with the Mini-Mental State Examination (MMSE) as a secondary outcome. However, the comparison only reached significance in weeks 8, 16 and 20. In addition, Tariot et al. (2001) [58] reported another secondary outcome, the Clinical Dementia Rating – Sum of Boxes Score (CDR-SoB), which showed a significant difference at the end of the treatment period in favour of donepezil. This difference was mainly due to cognitive worsening in the placebo group [58]. Only data from Burns et al. [57] was suitable for extraction and was used for quality of evidence rating. The quality of evidence of this outcome according to GRADE was very low.

#### BPSD

Tariot et al. (2001) measured BPSD as a primary outcome by using the Neuropsychiatric Inventory – Nursing Home Version (NPI-NH), which showed a non-significant difference between donepezil and placebo (MD 2.60; 95% CI: [− 2.67, 7.78]) [58]. There was no significant improvement in either group in the NPI-NH score. The quality of evidence of this outcome according to GRADE was low.

#### Adverse events

Both studies reported adverse events (AE) during the treatment period. When pooling data of both studies (n = 615) for the total number of AEs, a non-significant difference between AChEI (282/310, 91.0%) and placebo (279/305, 91.5%) was shown (RR 1.00; 95% CI: [0.95, 1.05]; I^2^ = 0%) [57, 58]. For gastrointestinal AEs, there was no significant difference between AChEI and placebo (RR 1.17; 95% CI: [0.83, 1.65]; I^2^ = 69%). In contrast, pooled neurological AEs (agitation, tremor, confusion, depression, aggression, vertigo, abnormal gait, dizziness) occurred significantly more often in the AChEI group (89/310, 28.7%) than in the placebo group (58/305, 19%; RR 1.53; 95% CI: [1.15, 2.03]; I^2^ = 44%). The quality of evidence for the total number of AEs according to GRADE was moderate.

#### Treatment tolerability

Treatment tolerability was assessed by using the total number of dropouts as a proxy. The pooled data showed no significant difference between AChEI and placebo (RR 0.87; 95% CI: [0.63, 1,19]; I^2^ = 0%) [57, 58]. The quality of evidence of this outcome according to GRADE was very low.

#### Deaths

**Fig. 3:**
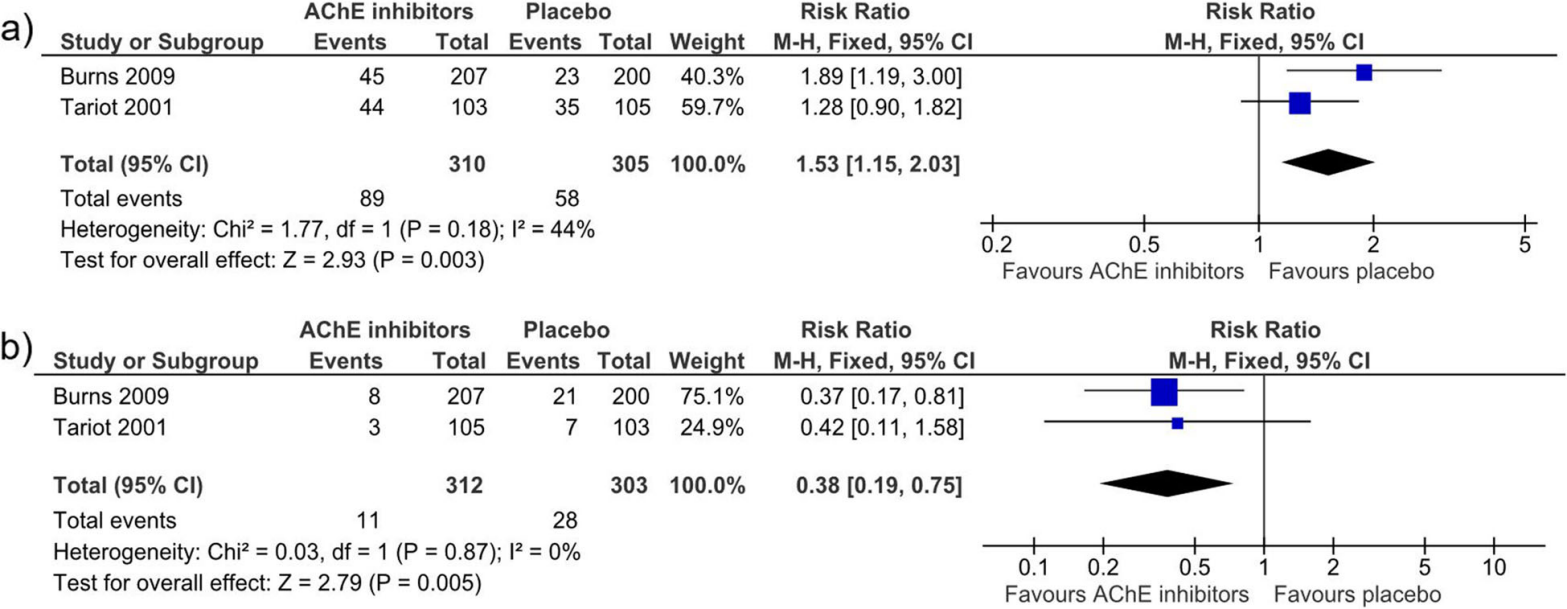
Forest plots of AChEI neurological AEs and deaths by Tariot et al. [58] and Burns et al. [57]. **a** Forest plot of the pooled neurological adverse events in Burns et al. and Tariot et al. **b** Forest plot of the pooled number of deaths in Burns et al. and Tariot et al. 95% CI = 95% confidence interval; AChE-I = acetylcholinesterase inhibitor; Chi^2^ = Chi^2^ test value to evaluate heterogeneity; df = degrees of freedom; fixed = fixed effects model; I^2^ = I^2^ test value to evaluate heterogeneity; IV = inverse variance; MD = mean difference; M-H = relative risk by Mantel-Haenszel; Neurolog. AEs = neurological adverse events; SD = standard deviation

The pooled data for the total number of deaths in both studies (N=61) indicated that there were significantly fewer deaths in the AChEI group than in the placebo group (RR 0.38; 95% CI: [0.19, 0.75]; I^2^ = 0%) [57, 58]. The quality of evidence of this outcome was low according to GRADE (Fig. 3).

### Antidepressants

Three RCTs investigating the efficacy and safety of antidepressants (clomipramine, fluoxetine, fluvoxamine) were included [56, 59, 65]. Unfortunately, one cross-over trial only reported outcomes graphically and statistical data were not suitable for extraction, except for data of AE data, that were included in the quantitative analysis [59]. None of the studies reported the number of deaths during the study period. All outcomes rated according to GRADE are summarized in evidence profiles (see Additional file 4).

#### Functional status

There was no significant difference between antidepressant and placebo/yokukansan in any of the studies as assessed by the Functional Independence Measure (FIM) as a secondary outcome. Petracca et al. (2001) reported no significant difference between fluoxetine and placebo (MD 2.70; 95% CI: [− 0.51, 5.91]); the quality of evidence of this outcome according to GRADE was low [56]. Teranishi et al. also showed no significant difference between fluvoxamine and yokukansan (MD − 7.71; 95% CI: [− 24.33, 8.91]); the quality of evidence of this outcome according to GRADE was low [65].

#### Cognitive function

Both Teranishi et al. and Petracca et al. (2001) reported a non-significant difference between fluoxetine/fluvoxamine and placebo/yokukansan, for the secondary outcome as assessed by the Mini-Mental State Examination (MMSE) (fluoxetine: MD − 0.8, 95% CI: [− 4.8, 3.2]; fluvoxamine: MD − 0.63, 95% CI: [− 3.34; 2.17]) [56, 65]. The quality of evidence of both outcomes according to GRADE was very low.

Petracca et al. (1996) reported non-significant changes in MMSE scores in the first treatment period: the clomipramine group showed a small improvement (~ 0.2 pt.), while the placebo group worsened by about 0.5 pt. in the MMSE [59]. Data was not suitable for extraction and was therefore not included in quantitative analysis and quality of evidence rating.

#### BPSD

Petracca et al. (2001) assessed BPSD with the Hamilton Rating Scale for Depression (HAM-D), as a primary outcome, and the Hamilton Rating Scale for Anxiety as a secondary outcome (HAM-A). Neither outcome measure showed significant differences between fluoxetine and placebo (MD HAM-A 0.80; 95% CI: [− 2.39, 3.99]) (MD HAM-D − 0.6; 95% CI: [− 3.99, 2.79]) [56]. Teranishi et al. showed no significant difference in the primary outcome of general BPSD symptoms assessed with the NPI-NH between fluvoxamine and yokukansan (MD − 2.16; 95% CI: [− 9.44, 5.12]) [65]. The quality of evidence of both outcomes according to GRADE was very low.

#### Clinical global impression

Petracca et al. (2001) reported the Clinical Global Impression – Improvement scale (CGI-I) scale as a continuous outcome (1 = very much improved; 7 = very much worsened). This primary outcome did not show significant differences between fluoxetine and placebo (MD − 0.30; 95% CI: [− 0.76, 0.16]) [56].

#### Adverse events

The pooled data from Petracca et al. (1996) and Petracca et al. (2001) showed that AEs were more frequent in patients treated with antidepressants (15/28, 53.6%) than in the placebo group (12/34, 35.3%); however, this finding did not reach significance (RR 1.31; 95% CI: [0.82, 2.09]; I^2^ = 0%) [56, 59]. Teranishi et al. also showed no significant difference between fluvoxamine and yokukansan regarding the total number of adverse events (RR 1.11; 95% CI: [0.8, 1.52]) [65]. Both outcomes’ quality of evidence according to GRADE was very low.

Teranishi et al. also assessed extra-pyramidal symptoms by the Drug-Induced Extra-Pyramidal Symptoms Scale (DIEPSS), which showed no significant difference between fluvoxamine and yokukansan (MD 0.15; 95% CI: [− 0.22, 0.52]) [65].

#### Treatment tolerability

The data from Petracca et al. (1996) and Teranishi et al. showed no significant differences in treatment tolerability for the comparison of antidepressants with placebo and yokukansan, respectively, as assessed by total numbers of dropouts (fluoxetine: RR 0.71, 95% CI: [0.15, 3.43]; fluvoxamine: RR 0.96, 95% CI: [0.15, 6.37]) [56, 65]. The quality of evidence of these outcomes according to GRADE was very low.

Petracca et al. (1996) (N=24) did not report dropouts separately for each treatment period but reported two dropouts in the placebo group and one dropout during clomipramine treatment [59].

### Anticonvulsants

Four RCTs investigating the efficacy and safety of anticonvulsants (carbamazepine, valproate) in frail older patients with AD and aggressive/agitated behaviour were included [60–63]. One small cross-over trial was not included in the quantitative analysis due to high risk of bias and insufficient reporting of statistical outcome data [62]. All outcomes rated according to GRADE are summarized in an evidence profile (see Additional file 5).

#### Functional status

All four studies assessed functional status by the Physical Self-Maintenance Scale, an ADL scale, as a secondary outcome. There was no significant difference in PSMS scores between anticonvulsants and placebo (MD 0.44; 95% CI: [− 0.33, 1.22]; I^2^ = 0%) [60, 61, 63]. The quality of evidence of this outcome was very low.

Also, the instrumental activities of daily living (IADL), which were assessed by Olin et al., showed no significant difference between carbamazepine and placebo (MD 0.40; 95% CI: [− 1.5, 2.3]) [60].

#### Cognitive function

The pooled data of three RCTs showed no significant differences between anticonvulsants and placebo in the secondary outcome of cognitive function assessed by the MMSE (MD 0.02; 95% CI: [− 1.44, 1.47]; I^2^ = 0%) [60, 61, 63]. The quality of evidence of this outcome was low. Tariot et al. (1994) also reported no significant differences in the MMSE between anticonvulsant and placebo [62].

#### BPSD

The total score of the Brief Psychiatric Rating Scale (BPRS), which was reported by three RCTs as the primary outcome, showed no significant difference between anticonvulsants and placebo (MD − 3.02; 95% CI: [− 7.62, 1.57]; I^2^ = 69%) [60, 61, 63]. The quality of evidence of this outcome was assessed as very low. However, two RCTs reported a significant reduction of the BPRS agitation factor in the group treated with anticonvulsants (MD − 2.07; 95% CI: [− 3.54, − 0.60]; I^2^ = 56%) [61, 63]. In addition, another two RCTs showed a significant reduction in the hostility factor of the BPRS in participants treated with carbamazepine (MD − 1.77; 95% CI: [− 2.54, − 0.99]; I^2^ = 0%) [60, 63]. When the carbamazepine data were pooled with valproate data from the study by Porsteinsson et al., the difference was no longer significant (MD − 1.21; 95% CI: [− 2.47, 0.05]; I^2^ = 72%) [61].

The Overt Aggression Scale (OAS), which was used in two RCTs as a secondary outcome, showed no significant difference in between anticonvulsants and placebo (MD − 2.36; 95% CI: [− 7.16, 2.45]; I^2^ = 74%) [61, 63]. Tariot et al. (1994) also reported no significant difference between carbamazepine and placebo as measured by the OAS [62].

Furthermore, secondary outcome measures for BSPD, such as the HAM-D in Olin et al. and the Cohen-Mansfield Agitation Inventory (CMAI) in Porsteinsson et al., showed no significant differences for the comparison between anticonvulsants and placebo (CMAI: MD − 2.20, 95% CI: [− 11.60, 7.20]; HAM-D: MD − 2.80, 95% CI: [− 6.17, 0.57]) [60, 61]. The Dementia Mood Assessment Scale (DMAS), which was used in Tariot et al. (1994) as a secondary outcome, also showed no significant difference between carbamazepine treatment and placebo [62].

#### Clinical global impression

All three RCTs included in quantitative analysis reported the clinical global impression (CGI-I) [60, 61, 63]. Olin et al. and Tariot et al. (1998) did report the CGI as a primary outcome, but Porsteinsson et al. did not specifically describe the CGI as a primary or secondary outcome. However, when including the data of Porsteinsson et al. the pooled outcome remained non-significant. This outcome was analysed as a dichotomous outcome and adjacent categories were combined into two groups: “worsening/no change” and “improvement”. The number of events equated to the number of participants whose clinical impression improved. The CGI-I showed no significant difference between treatment with anticonvulsants and placebo (RR 1.72; 95% CI: [0.76, 3.90]; I^2^ = 76%).

#### Adverse events

When pooling data of three RCTs with carbamazepine, there were more AEs in participants treated with anticonvulsants (39/64, 60.9%) than treated with placebo (24/64, 37.5%), but this difference did not reach significance (RR 1.49; 95% CI: [0.76, 2.93]; I^2^ = 67%) [60, 61, 63]. The quality of evidence of this outcome according to GRADE was very low. For valproate, Porsteinsson et al. reported significantly more valproate-treated participants with AEs (19/28, 67.9%) than placebo (9/28, 32.1%; RR 2.11; 95% CI: [1.16, 3.83]) [61].

Pooled data of the total number of serious and clinically significant AEs suggested an increased risk for AEs among those taking anticonvulsants (anticonvulsants 7/55, 12.7%; placebo 1/52, 0.02%; RR 4.81; 95% CI: [0.87, 26.56]; I^2^ = 0%) [61, 63]. Other trials did not report the total number of serious and clinically significant AEs [60, 62].

Two studies reported data on movement disorders (ataxia, postural instability, involuntary movement) and falls and showed a significant difference in favour of placebo (events on anticonvulsants: 42/55, 76.3%; events on placebo 26/52, 50%; RR 1.49; 95% CI: [1.13, 1.97]; I^2^ = 0%) [61, 63].

#### Treatment tolerability

All included studies reported the total number of dropouts [60, 61, 63]. Most of the dropouts in the placebo group were reported to be due to worsening of agitation and aggressive behaviour. There was no significant difference in treatment tolerability as assessed by the number of dropouts (anticonvulsants 7/63, 11.1%; placebo 8/64, 12.5%; RR 0.94; 95% CI: [0.36, 2.47]; I^2^ = 49%). The quality of evidence of this outcome was assessed as very low.

#### Death

Tariot et al. (1994) reported one death during the carbamazepine treatment period [62]. No other study reported any deaths.

### Antipsychotics

Two 3-arm studies investigating the efficacy and safety of antipsychotics were included in this review. Tariot et al. compared quetiapine and haloperidol with placebo in frail older patients with AD and psychotic symptoms [64]. Teranishi et al. compared risperidone with the Chinese herbal medicine yokukansan and fluvoxamine in AD patients with general BPSD [65]. Treatment effects of fluvoxamine are reported in the antidepressant section above. Due to different comparisons (placebo/yokukansan) and study populations (psychotic symptoms/general BPSD), separate evidence profiles were created. All rated outcomes according to GRADE are summarized in evidence profiles (see Additional file 6).

#### Functional status

Tariot et al. (2006) assessed functional status using the PSMS as a secondary outcome, which showed a significant worsening in subjects treated with haloperidol compared with placebo (MD 1.12; 95% CI: [0.33, 1.191]; Hedges’ g = 0.42), even though the mean daily dose (1.9 mg/day) and the median maximum dose (2.0 mg/day) were below the potentially inappropriate dose of > 2 mg/day as indicated in the PRISCUS list [34, 64]; in contrast, no significant decrease in functional status for quetiapine compared with placebo was shown (MD − 0.48; 95% CI: [− 1.33, 0.37]) [64]. Teranishi et al. also reported no significant changes in functional status (secondary outcome) as assessed with the FIM when comparing risperidone and placebo (MD − 1.15; 95% CI: [− 17.08, 14.78]) [65]. The quality of evidence of all outcomes regarding functional status according to MedQoL criteria was rated as very low.

In Tariot et al. (2006), the secondary outcome Multidimensional Observation Scale for Elderly Subjects (MOSES) - Social activities subscale also showed a significant worsening in subjects treated with haloperidol compared with placebo (MD 1.43; 95% CI: [0.18, 2,68]). Quetiapine-treated patients showed no significant differences in MOSES scores compared with placebo (MD − 0.07; 95% CI: [− 1.49, 1.35]) [64].

#### Cognitive function

None of the studies showed a significant difference in the secondary outcome, assessed with the MMSE scores when comparing antipsychotics and placebo/yokukansan (control groups) (haloperidol: MD − 0.16, 95% CI: [− 1.63, 1.31]; quetiapine: MD − 0.68, 95% CI: [− 1.92, 0.56]; risperidone: MD − 0.47; 95% CI: [− 3.04, 2.10]) [64, 65]. The quality of evidence for quetiapine and haloperidol was low, and the quality of evidence for risperidone was rated as very low.

#### BPSD

Even though Tariot et al. (2006) did not find any statistically significant changes in the primary outcome of total BPRS scores when comparing quetiapine and haloperidol with placebo^2^, analysis of the agitation factor subscale showed a significant improvement in subjects treated with quetiapine or haloperidol compared with placebo (haloperidol: MD − 1.41, 95% CI: [− 2.47, − 0.35]; quetiapine: MD − 1.18, 95% CI: [− 2.26, − 0.10]) [64]. In contrast, the BPRS anergia subscale showed no significant difference when comparing quetiapine and placebo (MD 0.40; 95% CI: [− 0.49, 1.29]), but there was a statistically significant worsening in the haloperidol group when compared with placebo-treated subjects (MD 1.94; 95% CI: [0.99, 2.89]). Another subscale analysis of the BPRS, thought disturbances, showed no statistically improvement or worsening when comparing antipsychotics with placebo (haloperidol: MD − 0.31, 95% CI: [− 1.22, 0.60]; quetiapine: MD − 0.43; 95% CI: [− 1.36, 0.50]).

Additionally, Tariot et al. (2006) administered the NPI-NH as a secondary outcome and found a significant improvement in the NPI-NH total score in subjects treated with haloperidol compared with placebo (MD − 1.82; 95% CI: [− 3.51, − 0.13]). Quetiapine showed no significant difference in the NPI-NH total score compared with placebo (MD − 0.03; 95% CI: [− 1.79, 1.73]) [64]. Both quetiapine and haloperidol showed non-significant improvements in the NPI-NH agitation subitem analysis for antipsychotics compared with placebo (haloperidol: MD − 0.94, 95% CI: [− 2.23, 0.35]; quetiapine: MD − 1.14; 95% CI: [− 2.51, 0.23]). Teranishi et al. used the NPI-NH as the primary outcome to measure BPSD and reported no significant differences in the NPI-NH total score when comparing risperidone and yokukansan (MD 2.72; 95% CI: [− 3.34, 8.87]) [65].

The quality of evidence for BPSD as assessed with the NPI-NH was low for quetiapine and haloperidol, and the quality of evidence for risperidone was rated as very low according to GRADE.

#### Clinical global impression

Tariot et al. (2006) found no statistically significant differences in the primary outcome of clinical global impression of illness severity score comparing quetiapine and haloperidol with placebo (quetiapine: MD − 0.13, 95% CI: [− 0.42, 0.16]; haloperidol: MD − 0.05, 95% CI: [− 0.33, 0.23]) [64].

#### Adverse events

Tariot et al. (2006) used the Abnormal Involuntary Movement Scale (AIMS) and the Simpson-Angus Scale (SAS) to assess extrapyramidal symptoms, but none of those showed any significant differences between quetiapine and placebo (AIMS: MD 0.12, 95% CI: [− 0.56, 0.80]; SAS: MD 0.00, 95% CI: [− 1.14, 1.14]). In contrast, Tariot et al. (2006) found a significant worsening in extrapyramidal symptoms as assessed with the SAS in subjects treated with haloperidol compared with placebo (MD 2.72; 95% CI: [1.36, 4.08]) and a nearly significant difference in the AIMS score compared with placebo (MD 0.58; 95% CI: [− 0.10, 1.26]) [64]. The quality of evidence regarding extrapyramidal symptoms assessed with the SAS for haloperidol and quetiapine was low and very low, respectively.

Teranishi et al. also assessed extrapyramidal symptoms by using the Drug-Induced Extra-Pyramidal Symptoms Scale (DIEPPS), which showed a significant worsening in risperidone treated subjects compared with yokukansan (MD 0.87; 95% CI: [0.43, 1.31]) [65]. The quality of evidence for this outcome was rated as low.

Both studies reported on AEs and severe AEs. Teranishi et al. did not find any statistically significant differences between risperidone and yokukansan in the total number of AEs (RR 1.20; 95% CI: [0.90, 1.61]) and serious adverse events (SAE; as described by the authors as fall with contusion, oversedation, swallowing difficulty, stridor or sudden death; RR 1.73; 95% CI: [0.46, 6.52]) [65]. Tariot et al. (2006) also found no significant differences between haloperidol/quetiapine and placebo in the total number of severe AEs (haloperidol: RR 1.30, 95% CI: [0.64, 2,64]; quetiapine: RR 0.90, 95% CI: [0.41, 1.98]) [64]. In addition, the total number of falls and fractures in subjects treated with antipsychotics were not significantly different to placebo/yokukansan (haloperidol: RR 0.98, 95% CI: [0.76, 1.44], P = 0.93; quetiapine: RR 0.86, 95% CI: [0.57, 1.29]; risperidone: RR 0.52, 95% CI: [0.05, 5.39]). However, the further analysis of certain AEs found some significant differences between antipsychotics and placebo: neurological AEs, such as somnolence, agitation, abnormal gait, insomnia and convulsion were significantly more frequent in subjects treated with haloperidol (62/94, 65.9%) than in the placebo group (29/98, 29.6%; RR 2.23, 95% CI: [1.59, 3.13]). Furthermore, Tariot et al. (2006) reported significantly more infections (not further specified) in the quetiapine group (13/91, 14.3%) than in the placebo group (5/98, 5.1%; RR 2.69, 95% CI: [1.00, 7.23]).

#### Treatment tolerability

Neither study showed significant differences in dropout rates when comparing antipsychotics with placebo/yokukansan (haloperidol: 39/94, 41.5%; RR 1.14, 95% CI: [0.80, 1.63]; quetiapine: 29/91, 31.9%; RR 0.88, 95% CI: [0.59, 1.30]; risperidone: 2/28, 7.1%; RR 1.93, 95% CI: [0.19, 20.05]; placebo: 36/99, 36.4%; yokukansan: 1/27, 3.7%) [64, 65]. The quality of evidence of this outcome was very low for all interventions according to GRADE.

#### Deaths

There were also no statistically significant findings regarding the total number of deaths during the study period (haloperidol: 7/94, 7.4%; RR 1.82, 95% CI: [0.55, 6.03]; quetiapine: 2/91, 2.2%; RR 0.54, 95% CI: [0.10, 2.87]; risperidone: 1/27, 3.7%; RR 3.0, 95% CI: [0.13, 70.53]; placebo: 4/98, 4.1%; yokukansan: 0/27, 0%) [64, 65]. The quality of evidence of this outcome according to GRADE was very low for all interventions.

## Discussion

This systematic review evaluated the efficacy and safety of pharmacotherapy for AD and BPSD in frail older patients. Despite the increasing number of publications on frailty [19, 68] and the call by several institutions to include more frail older patients in clinical trials [11, 31], only ten eligible studies, mainly of small study population size, were identified. Many of the screened studies were excluded due to a lack of functional status assessment or frailty evaluation, and it was therefore not possible to classify included patients as frail or non-frail. However, we were able to identify two RCTs focusing on AChEIs, four on anticonvulsants, one on antipsychotics, two on antidepressants, and one triple-arm study investigating one antidepressant and one antipsychotic medication. Unfortunately, quality of life (QoL), which is considered an important outcome in AD trials by the EMA, was not assessed in any of the included studies [44]. For all outcomes of the included RCTs quality of evidence was mostly of (very) low quality, mainly due to the small study size. Overall, the small number of eligible studies identified in our literature review is in line with previous publications showing that frail elderly patients are often underrepresented in clinical trials [69–71].

In general, our systematic review suggested minimal positive effects of pharmacotherapy for AD and associated BPSD, and indicated possible harms with some treatments. Our data analysis suggested that frail older patients treated with AChEIs had slight improvements in cognition and that AChEIs were generally tolerated well in frail older patients. Antidepressants did not show any significant improvements in depressive symptoms or BPSD in general but were well-tolerated. RCTs of anticonvulsants showed some significant improvement in certain BPSD items (hostility, agitation), but no significant reduction of total BPSD scores. Antipsychotics, especially haloperidol, showed some significant efficacy on certain BPSD items (anergia, agitation) and NPI-NH total score, but showed no effect on clinical global impression ratings.

There were also possible negative treatment effects shown in frail older patients. Haloperidol-treated subjects scored significantly worse in ADLs and experienced more neurological AEs. Furthermore, haloperidol- and risperidone-treated frail older patients experienced significantly more EPMS and significantly more falls and fractures. Furthermore, there were significantly more falls and movement disorders in anticonvulsant-treated frail older patients and a significantly higher number of valproate-treated patients who experienced at least one AE.

### Acetylcholinesterase inhibitors

We reviewed 2 studies of AChEIs by Tariot et al. (2001) and Burns et al. [57, 58]. In both studies, it was planned to treat patients with the recommended maximum dose if possible; average daily doses were above the minimum effective daily dose. Neither trial showed significant changes in functional status as assessed with ADL measures (MDS-ADL, PSMS) when comparing AChEIs with placebo. These findings contrast with those of larger meta-analyses of AChEI in AD by the Cochrane Collaboration and the IQWiG, which indicated that AChEs showed significant superiority over placebo treatment regarding ADL; however, these studies did not specifically focus on frail older patients [72, 73]. The efficacy of donepezil in the treatment of BPSD in younger patients is also debatable: A Cochrane review [74] from 2006 of donepezil treatment in patients with AD found only small significant effects of donepezil on BPSD, and a review by the IQWiG of donepezil treatment in patients with AD showed no relevant benefit of donepezil [73]. Tariot et al. (2001) also found no significant treatment effect of donepezil on BPSD [58]. The small magnitude of the treatment effect regarding cognitive function in Burns et al. [57] (Hedges’ g = 0.36) was similar to previous studies with other AChEIs in severe AD in younger patients [75, 76].

Importantly, in the two included studies, there were significantly fewer deaths in the AChEI groups than in the placebo groups. Even if the quality of evidence was low—mainly due to the small number of participants—reduced mortality for AD patients treated with AChEIs has also been shown in previous systematic reviews evaluating efficacy, safety and cardiovascular outcomes in AD patients treated with AChEI involving larger patient numbers [77, 78]. The decreased mortality in other studies was mainly accounted for by a reduction in cardiovascular events [79]. This finding is of questionable benefit in frail older populations, as life extension is usually not a treatment aim and subjective well-being and other palliative care goals are more desirable.

A Cochrane review evaluating efficacy and safety of AChE inhibitors in AD patients, which did not specifically focus on frail older patients, indicated significantly more dropouts and adverse events in subjects treated with AChEIs [72]. In contrast, the two studies we analysed suggested that according to MedQoL criteria treatment with AChEIs was generally well tolerated in frail older patients. These findings with respect to total number of AEs are somewhat reassuring, since frail older patients might have been expected to be more vulnerable to adverse effects than younger patient populations in other AD clinical trials [10, 14, 15]. However, neurological AEs (agitation, tremor, confusion, depression, aggression, vertigo, abnormal gait, dizziness) were significantly more frequent in patients receiving AChEIs.

### Antidepressants

We found two RCTs investigating antidepressants (clomipramine, fluoxetine) in patients with AD and depression [56, 59]. One 8-week RCT also investigated the antidepressant fluvoxamine compared with yokukansan in patients with severe AD, vascular dementia and dementia with Lewy bodies and general BPSD in a Japanese psychiatric hospital [65].

In the included RCTs, there was no significant improvement in depressive symptoms/BPSD in frail older patients. In younger AD patients, the current evidence generally supports the use of antidepressants (i.e. SSRIs for treatment of depression [80]); however, evidence for their efficacy in AD-related BPSD is contradictory [56, 59, 65]. A Cochrane review from 2018 of depression in dementia that did not specifically focus on frail older patients showed no significant difference in reduction of depression scores between antidepressant and placebo, but significant superiority of antidepressants in depression remission rates [81]. A systematic review of antidepressants in BPSD found some efficacy of antidepressants in the treatment of BPSD [82]. These differences could be due to the heterogeneity in patient populations and the use of different antidepressants. In terms of functional status and cognition, none of the three studies of antidepressants included in our systematic review showed significant benefits in functional status or cognitive function compared with placebo or yokukansan. This result is in line with a Cochrane review of antidepressants in AD from 2018, which also did not show a cognitive or functional benefit of antidepressants [81]. However, some smaller reviews have found that antidepressants (other than tricyclics, which could decrease cognitive function [83]) may reduce cognitive decline in the course of AD [84, 85]. The duration of included studies was probably too short to detect any protective effects on cognition.

All three studies indicated that antidepressants were generally well tolerated, although the number of patients was too small to draw firm conclusions. A similar result was obtained in a systematic review including five studies of antidepressants in AD [83]. In contrast, a larger meta-analysis from 2018 including a total of 1592 AD patients found a significantly higher number of AEs and discontinuations in patients treated with antidepressants than with placebo [81]. Given the unclear state of the evidence, an individual benefit-risk assessment and the preferred use of non-pharmacological interventions is recommended.

### Anticonvulsants

Taken together, the four studies included in our review did not provide clear evidence of the effect of anticonvulsants on BPSD. When all 3 studies with carbamazepine were included, the effect on agitation and hostility remained significant in favour of carbamazepine. However, when the valproate study was included, neither the findings for BPRS nor hostility remained significant. This result is in line with a meta-analysis of valproate, which also found no significant treatment effect in patients with AD [86]. However, there remains the possibility that carbamazepine is effective in agitated and aggressive behaviour in patients with dementia [87, 88]. The evidence is complicated by heterogeneity when data from the four studies were pooled, as there were relevant differences in the study treatments (carbamazepine vs. valproate) and in the patient populations (e.g. Olin et al. included patients who had treatment-resistant symptoms when previously treated with antipsychotics) [60].

The four studies we reviewed showed no significant worsening in cognition over the course of treatment [89, 90]. Conversely, a study in non-frail patients demonstrated more rapid cognitive deterioration and increased brain atrophy in long-term use of valproate in patients with AD [91].

There were significantly more patients treated with valproate who experienced at least one adverse event in comparison with placebo [61]. Other reviews of pharmacotherapy of agitation and aggression in dementia with anticonvulsants have come to similar conclusions [87, 92]. While Tariot et al. (1998) had significantly more adverse events in the carbamazepine group, Olin et al. showed no significant difference between the carbamazepine and placebo groups [60, 63]. This may be due to the higher average age of participants in Tariot et al. (1998), but given the small study size, this cannot be definitively determined [63]. Furthermore, pooled data of movement disorders and falls showed significantly more events in participants treated with anticonvulsants compared with placebo [61, 63]. Two other reviews regarding the use of antiepileptic drugs in older patients also showed an association between anticonvulsant treatment and falls in older patients [93, 94].

It is not possible to make specific recommendations regarding the use of anticonvulsants in frail older patients with dementia and aggressive/agitated behaviour. Potential adverse outcomes should be carefully evaluated when the use of carbamazepine is considered [95]. At least theoretically, treatment with anticonvulsants might allow reduction in the use of antipsychotics, which are also often used in agitated and aggressive dementia patients and have their own set of problems [96, 97]. Further studies are urgently needed.

### Antipsychotics

In the study by Tariot et al. 2006, both quetiapine and haloperidol showed small but significant reductions in the BPRS agitation factor scores compared with placebo (haloperidol Hedges’ g = 0.39, quetiapine Hedges’ g = 0.32). Haloperidol was also shown to have a small but significant superiority over placebo in reduction of the NPI-NH total score (Hedges’ g = 0.31) [64]. Other systematic reviews of atypical antipsychotics and haloperidol in dementia have also shown a small but significant reduction of agitated behaviour after treatment with atypical antipsychotics [98] and a reduction of aggressive behaviour after treatment with haloperidol [99] (Hedges’ g ≥ 0.2). The effect of the antipsychotic treatment on the overall clinical picture appears to be minimal. In Teranishi et al., there were no significant changes in overall BPSD between risperidone and yokukansan [65]. This could be due to lack of efficacy, or also due to the heterogenous study population, which in addition to patients with AD also included participants with dementia with Lewy bodies, vascular dementia and severe dementia (average MMSE 5 pt.).

Even though none of the included studies showed significant changes in cognitive function, there are a few studies that have reported a negative impact of antipsychotics on cognitive function in AD patients [100–102]. However, any cognitive decline due to antipsychotic treatment in the study by Tariot et al. 2006 was of smaller magnitude than reported in other clinical trials [103], and psychotic symptoms themselves seem to be generally associated with more rapid cognitive deterioration in AD [104, 105]. Causality therefore cannot be established.

In terms of adverse events, previous studies in dementia patients have shown that haloperidol and risperidone are associated with more frequent and severe EPMS [98, 99, 102]; haloperidol may be more problematic for dementia patients than risperidone [106, 107]. Including both studies in frail older patients according to MedQoL criteria, we found significant differences in EPMS scores for haloperidol- and risperidone-treated subjects [64, 65]. There was also a significant worsening in functional status in frail older subjects treated with haloperidol [64]. Overall, Tariot et al. (2006) reported significantly more neurological adverse events in haloperidol-treated patients than in patients treated with placebo or quetiapine [64]. However, current evidence also shows that even patients with dementia and AD treated with atypical antipsychotics, which were not specifically classified as frail, have significantly higher numbers of neurological adverse events in comparison with placebo [98, 102].

Treatment tolerability assessed by on the basis of the total number of dropouts suggested that antipsychotics were well-tolerated, and no significant higher number of deaths in included frail older patients was found.

In contrast, larger RCTs and meta-analyses have shown a significantly higher rate of cerebrovascular events, dropouts and deaths for patients with dementia treated with antipsychotics [99, 102, 108–110], although increased mortality may not be significant when confounding factors such as age, cognitive impairment and the occurrence of BPSD are taken into consideration [111, 112]. The number of participants in the included studies was probably not sufficient to show those negative treatment effects.

Due to the small number of studies and the small study populations, all outcomes’ quality of evidence was rated as low to very low according to GRADE. Known adverse effects of antipsychotics (EPMS, infection, possible cognitive decline) were also demonstrated in the two studies we evaluated. Antipsychotics should be avoided if possible according to the Beers’ criteria [113]. Therefore, antipsychotics may also be inappropriate for the use in frail older patients. According to German national guidelines, due to the unfavourable side effect profile, antipsychotics should only be used in severe BPSD when non-pharmacological interventions are not sufficient. Their use should be re-evaluated on a regular basis [114], because some evidence suggests that discontinuation may not significantly worsen BPSD and may decrease the risk of mortality [115, 116].

## Strengths and limitations

To our knowledge, this is the first systematic review of RCTs regarding drug therapy of AD and BPSD in frail older patients. In an exhaustive literature research, approximately 45,000 records were screened. Unfortunately, only ten studies were eligible for inclusion in this review, and most of them had only small numbers of patients. Most RCTs did not list any data about functional status or frailty, so that it was not possible to ascertain whether study patients met our inclusion criteria. Because of the small number of eligible RCTs, quality of evidence according to GRADE was mostly (very) low. Therefore, no specific treatment recommendations can be made.

We excluded physical frailty assessments that consisted mainly of cognitive items, because some assessments, such as the ADL, could also indicate impairment in severe dementia even if there was no clear physical frailty. However, some patients with physical frailty could also have been inadvertently excluded. Furthermore, despite our attempt to use only “non-cognitive” functional measures to assess frailty, cognition might have also have had an indirect impact on the assessments. This might especially apply to later stages of AD, when motor function could potentially also be affected.

One of the difficulties in our research was reliably identifying studies that included physically frail patients, because physical frailty assessments are seldom performed or reported in RCTs. It is possible that frail patients were included in some additional studies, but that their data were not reported separately, thereby obscuring the effects of pharmacotherapy on this specific group. In future RCTs in older patients with AD, the use of physical performance-based assessments used in general geriatric practice, such as handgrip strength and gait speed or the Short-Physical Performance Battery (SPPB) should be strongly considered [117–119], so that the data of frail patients can be analysed as a subgroup. Such efforts would greatly improve the reliability of recommendations for pharmacological therapy of physically frail patients.

## Conclusion

Although today’s frailty definition was introduced approximately 30 years ago and despite the recommendation by regulatory authorities that frail older patients be included in clinical trials, an exhaustive literature assessment identified only a few small studies that specifically included patients with physical frailty or significant functional impairment. Physically frail older patients appear to be severely underrepresented in clinical trials, although they represent the major users of many pharmaceuticals [8–10]..

### Implications for practice

The available data suggested small but significant improvements of cognition in AChEI-treated patients and good treatment tolerability. Antidepressants did not show any significant improvements in depressive symptoms or BPSD in general. Both antipsychotics and anticonvulsants demonstrated significant improvements in certain BPSD items, but also higher rates of adverse events. The overall data was of low to very low quality due to the small numbers of included patients, and therefore it is difficult to make treatment recommendations with any degree of confidence. This review highlights the overall lack of evidence regarding the efficiency of pharmacotherapy on AD and BPSD in frail older and significantly functional impaired patients. In the absence of better evidence, in general, an individual risk-benefit analysis should be performed, and pharmacological treatment should only be started if non-pharmacological interventions are insufficient. Frail older patients appear to possibly be more vulnerable to drug-induced AEs, and therefore, regular re-evaluation of drug therapy is beneficial [15].

### Implication for research

Due to demographic changes, physical frailty will become more important in medical care, and it should be taken into greater account in study planning and reporting—for example, by implementing a standardized procedure in the CONSORT guidelines [41, 120]. One possible approach was demonstrated in a study that investigated the safety and efficacy of nilvadipine in patients with mild to moderate AD and included frail older patients in a planned substudy [121]. More clinical trials that include measures of physical function or frailty such as physical performance-based assessments and ADL scales are urgently needed. It would also be helpful if trials that include frail patients—even if this is only a subgroup—could separately identify these patients in the study results.

## Supplementary Information

**Additional file 1.**

**Additional file 2.**

**Additional file 3.**

**Additional file 4.**

**Additional file 5.**

**Additional file 6.**

**Additional file 7.**

## Data Availability

The datasets used and analysed during the current review are available from the corresponding author on reasonable request.

## Abbreviations

95% CI: 95% confidence interval
AChEI: Acetylcholinesterase inhibitor
ADL: Activities of daily living
AE: Adverse event
AIMS: Abnormal Involuntary Movement Scale
BPRS: Brief Psychiatric Rating Scale
BPSD: Behavioural and psychological symptoms of dementia
CERAD BRSD: Behavior Rating Scale for Dementia of the Consortium to Establish a Registry for Alzheimer’s Disease
CDR-SoB: Clinical Dementia Rating – Sum of Boxes Score
CENTRAL: Cochrane Central Register of Controlled Trials
CGI-I: Clinical Global Impression (of Improvement)
CMAI: Cohen-Mansfield Agitation Inventory
DIEPSS: Drug-Induced Extra-Pyramidal Symptom Scale
DMAS: Dementia Mood Assessment Scale
DSM: Diagnostic and Statistical Manual of Mental Disorders
EMA: European Medicines Agency
EPOC: The Effective Practice and Organisation of Care Group
FIM: Functional Independence Measure
GRADE: Grading of Recommendations, Assessment, Development and Evaluation
Ham-A: Hamilton Rating Scale for Anxiety
Ham-D: Hamilton Rating Scale for Depression
IADL: Instrumental activities of daily living
ICD: International Classification of Diseases
IQWiG: German Institute for Quality and Efficiency in Health Care
MCI: Mild cognitive impairment
MD: Mean difference
MDS: Minimum Data Set
MDS-ADL: Minimum Data Set – Activities of Daily Living
MedQoL: Medication and Quality of Life in frail older persons Research Group
MID: Minimally important difference
MMSE: Mini-Mental State Examination
MOSES: Multidimensional Observation Scale for Elderly Subjects
NINCDS-ADRDA: National Institute of Neurological and Communicative Disorders and Stroke and the Alzheimer's Disease and Related Disorders Association
NPI-NH: Neuropsychiatric Inventory – Nursing Home Version
OAS: Overt Aggression Scale
PRISMA: Preferred Reporting Items for Systematic Reviews and Meta-Analyses
PSMS: Physical Self-Maintenance Scale
RCT: Randomized controlled trial
RevMan: Review Manager
RR: Risk ratio
SAS: Simpson-Angus Scale
SD: Standard deviation
SIB: Severe Impairment Battery
SMD: Standardized mean difference
SoF: Summary of findings

## Footnotes

1 Covidence is the primary screening and data extraction tool for Cochrane authors.

2 Haloperidol: MD − 0.39; 95% CI: [− 3.43, 2.65]. Quetiapine: MD − 2.32; 95% CI: [− 5.41, 0.77]
