# Supplementary material for: Efficacy and safety of pharmacotherapy for Alzheimer’s disease and for behavioural and psychological symptoms of dementia in older patients with moderate and severe functional impairments: a systematic review of controlled trials
Source: Alzheimers Res Ther. 2021 Jul 16;13:131. doi: 10.1186/s13195-021-00867-8 (PMC8285815; doi:10.1186/s13195-021-00867-8)
Supplement: Supplementary file 1 — Additional file 1. [file 13195_2021_867_MOESM1_ESM.docx]

Additional file 1

Minimally important difference (MID) for GRADE ratings

| **Score** | **MID** |
| --- | --- |
| Brief Psychiatric Rating Scale (BPRS) | 6.4-7.6 points |
| Drug-Induced Extrapyramidal Symptom Scale (DIEPSS) | 2 points |
| Functional Independence Measure (FIM) | 22 points |
| Hamilton Rating Scale for Depression 17-item version (HAM-D) | 3 points |
| Minimum Data Set – Activities of Daily Living (MDS-ADL) | 1 point |
| Mini Mental State Examination (MMSE) | 3 points |
| Neuropsychiatric Inventory – Nursing Home Version (NPI-NH) | 8 points |
