# Supplementary material for: Efficacy and safety of pharmacotherapy for Alzheimer’s disease and for behavioural and psychological symptoms of dementia in older patients with moderate and severe functional impairments: a systematic review of controlled trials
Source: Alzheimers Res Ther. 2021 Jul 16;13:131. doi: 10.1186/s13195-021-00867-8 (PMC8285815; doi:10.1186/s13195-021-00867-8)
Supplement: Supplementary file 2 — Additional file 2. [file 13195_2021_867_MOESM2_ESM.docx]

Additional file 2

Physical frailty evaluation and categorization according to MedQoL criteria.

|  | **Study** | **Assessment for the evaluation of functional status** | **Functional status at baseline stratified by groups, mean (SD)** | **Categorization according to functional status by MedQoL criteria** |
| --- | --- | --- | --- | --- |
| **AChEI** | *Burns et al. 2009* | Minimum Data Set – Activities of Daily Living | Galantamine: 11.9 (7.6)  Placebo: 12.6 (7.5) | Significantly impaired/partially dependent |
|  | *Tariot et al. 2001* | Physical Self-Maintenance Scale (ADLs) | Donepezil: 15.4 (4.9)  Placebo: 14.7 (5.0) | Significantly impaired/partially dependent |
| **Antidepressants** | *Petracca et al. 1996*  (cross-over trial) | Functional Independence Measure | C-P: 67.9 (2.9)  P-C: 64.7 (11.0) | Significantly impaired/partially dependent |
|  | *Petracca et al. 2001* | Functional Independence Measure | Fluoxetine: 68.5 (3.4)  Placebo: 64.2 (8.9) | Significantly impaired/partially dependent |
| **Anticonvulsants** | *Olin et al. 2001* | Physical Self-Maintenance Scale (ADLs) | Carbamazepine: 15.4 (5.4)  Placebo: 14.3 (3.8) | Significantly impaired/partially dependent |
|  | *Porsteinsson et al. 2001* | Physical Self-Maintenance Scale (ADLs) | Valproate: 15.4 (4.4)  Placebo: 14.3 (4.8) | Significantly impaired/partially dependent |
|  | *Tariot et al. 1994*  (cross-over trial) | Physical Self-Maintenance Scale (ADLs) | All participants: 16.3 (5.4) | Significantly impaired/partially dependent |
|  | *Tariot et al. 1998* | Physical Self-Maintenance Scale (ADLs) | Carbamazepine: 16.3 (4.2)  Placebo: 14.0 (4.7) | Significantly impaired/partially dependent |
| **Antipsychotics** | *Tariot et al. 2006* | Physical Self-Maintenance Scale (ADLs) | Haloperidol: 17.81 (4.63)  Quetiapine: 17.66 (5.51)  Placebo: 17.32 (5.64) | Significantly impaired/partially dependent |
| **Antipsychotics/**  **antidepressants** | *Teranishi et al. 2013* | Functional Independence Measure | Fluvoxamine: 60.92 (29.39)  Risperidone: 64.40 (29.50)  Yokukansan: 61.23 (29.05) | Severely  Impaired/Disabled/  Mostly or Totally  Dependent |
| ADLs, activities of daily living; C-P, Clomipramine-Placebo-Group; FIM, Functional Independence Measure; MDS-ADL, Minimum Data Set – Activities of Daily Living; P-C, Placebo-Clomipramine-Group; PSMS, Physical Self-Maintenance Scale.  Categories of functional status according to MedQoL criteria: (I) functionally independent, (II) functionally slightly impaired, (III) functionally significantly impaired/partially dependent and (IV) functionally severely impaired/disabled/mostly or totally dependent. | | | | |
