# Supplementary material for: Efficacy and safety of pharmacotherapy for Alzheimer’s disease and for behavioural and psychological symptoms of dementia in older patients with moderate and severe functional impairments: a systematic review of controlled trials
Source: Alzheimers Res Ther. 2021 Jul 16;13:131. doi: 10.1186/s13195-021-00867-8 (PMC8285815; doi:10.1186/s13195-021-00867-8)
Supplement: Supplementary file 3 — Additional file 3. [file 13195_2021_867_MOESM3_ESM.docx]

Additional file 3

AChEI compared with placebo in older patients with AD: GRADE evidence profile.

| **Certainty assessment** | | | | | | **№ of patients** | | **Effect** | | **Certainty** |
| --- | --- | --- | --- | --- | --- | --- | --- | --- | --- | --- |
| **№ of studies** | **Study design** | **Risk of Bias** | **Inconsistency** | **Indirectness** | **Imprecision** | **AChEI** | **Placebo** | **Relative**  **[95% CI]** | **Absolute**  **[95% CI]** |  |
| *Functional status (assessed with: MDS-ADL)* | | | | | | | | | | |
| 1 | RCT | Serious ^a^ | Not serious | Not serious | Very serious ^b,c^ | 186 | 178 | - | MD 0.4 pt. lower  [-1.32, 0.52] | ⨁◯◯◯  VERY LOW |
| *Cognitive function (assessed with: SIB)* | | | | | | | | | | |
| 1 | RCT | Serious ^a^ | Not serious | Not serious | Very serious ^b,c^ | 186 | 178 | - | MD 5.2 pt.  higher  [2.24, 8.16] | ⨁◯◯◯  VERY LOW |
| *BPSD (assessed with: NPI-NH)* | | | | | | | | | | |
| 1 | RCT | Serious ^a^ | Not serious | Not Serious | Serious ^b^ | 103 | 105 | - | MD 2.6 pt.  higher  [-2.67, 7.87] | ⨁⨁◯◯  LOW |
| *Adverse events* | | | | | | | | | | |
| 2 | RCT | Serious ^a^ | Not serious | Not serious | Not serious | 282/310 (91.0%) | 279/305 (91.5%) | RR 1.00  [0.95, 1.05] | 0 fewer per  1.000 (from 46 fewer to 46 more) | ⨁⨁⨁◯  MODERATE |
| *Death* | | | | | | | | | | |
| 2 | RCT | Serious ^a^ | Not serious | Not serious | Serious ^d^ | 11/312 (3.5%) | 28/303 (9.2%) | RR 0.38  [0.19, 0.75] | 57 fewer per  1.000 (from 75 fewer to 23 fewer) | ⨁⨁◯◯  LOW |
| Treatment tolerability (assessed by proxy with total numbers of dropouts) | | | | | | | | | | |
| 2 | RCT | Serious ^a^ | Not serious | Serious ^e^ | Very serious ^c,d^ | 58/310 (18.7%) | 66/305  (21.6%) | RR 0.87  [0.63, 1.19] | 28 fewer per  1.000 (from 80 fewer to 41 more) | ⨁◯◯◯  VERY LOW |
| *Quality of life:* not reported / not assessed. | | | | | | | | | | |
| AChEI: Acetylcholinesterase inhibitors; BPSD: Behavioural and psychological symptoms of dementia; CI: Confidence interval; MD: Mean difference; MDS-ADL: Minimum Data Set – Activities of Daily Living; NPI-NH: Neuropsychiatric Inventory – Nursing Home Version; RR: Risk ratio; SIB: Severe Impairment Battery.  **Explanations:** a. Three out of 6 risk of bias domains with unclear risk; b. Less than 400 participants; c. 95% CI includes probably relevant and irrelevant effects; d. Does not match optimal information size (OIS) criterion; e. Treatment tolerability assessed by proxy measure. | | | | | | | | | | |
