# Supplementary material for: Efficacy and safety of pharmacotherapy for Alzheimer’s disease and for behavioural and psychological symptoms of dementia in older patients with moderate and severe functional impairments: a systematic review of controlled trials
Source: Alzheimers Res Ther. 2021 Jul 16;13:131. doi: 10.1186/s13195-021-00867-8 (PMC8285815; doi:10.1186/s13195-021-00867-8)
Supplement: Supplementary file 4 — Additional file 4. [file 13195_2021_867_MOESM4_ESM.docx]

Additional file 4

Antidepressants (fluoxetine, clomipramine) compared with placebo in older patients with AD: GRADE evidence profile.

| **Certainty assessment** | | | | | | **№ of patients** | | **Effect** | | **Certainty** |
| --- | --- | --- | --- | --- | --- | --- | --- | --- | --- | --- |
| **№ of studies** | **Study design** | **Risk of Bias** | **Inconsistency** | **Indirectness** | **Imprecision** | **Antidepressants** | **Placebo** | **Relative**  **[95% CI]** | **Absolute**  **[95% CI]** |  |
| *Functional status (assessed with: FIM)* | | | | | | | | | | |
| 1 | RCT | Serious ^a^ | Not serious | Not serious | Serious ^b^ | 17 | 24 | - | MD 2.7 pt. higher  [-0.51, 5.91] | ⨁⨁◯◯  LOW |
| *Cognitive function (assessed with: MMSE)* | | | | | | | | | | |
| 1 | RCT | Serious ^a^ | Not serious | Not serious | Very serious ^b,c^ | 17 | 24 | - | MD 0.8 pt. lower  [-4.8, 3.2] | ⨁◯◯◯  VERY LOW |
| *BPSD (assessed with: HAM-D)* | | | | | | | | | | |
| 1 | RCT | Serious ^a^ | Not serious | Not serious | Very serious ^b,c^ | 17 | 24 | - | MD 0.6 pt. lower  [-3.99, 2.79] | ⨁◯◯◯  VERY LOW |
| *Adverse events* | | | | | | | | | | |
| 2 | RCT | Serious ^a^ | Not serious | Not serious | Very serious ^c,d^ | 15/28 (53.6%) | 12/34 (35.3%) | RR 1.31  [0.82, 2.09] | 109 more per 1.000 (from 64 fewer to 385  more) | ⨁◯◯◯  VERY LOW |
| Treatment tolerability (assessed by proxy with total numbers of dropouts) | | | | | | | | | | |
| 1 | RCT | Serious ^a^ | Not serious | Serious ^e^ | Very serious ^d,f^ | 2/17 (11.8%) | 4/24 (16.7%) | RR 0.71  [0.15, 3.43] | 48 fewer per  1.000 (from  142 fewer to  405 more) | ⨁◯◯◯  VERY LOW |
| *Death, quality of life:* not reported / not assessed. | | | | | | | | | | |
| BPSD: Behavioural and psychological symptoms of dementia; CI: Confidence interval; FIM: Functional Independence Measure; HAM-D: Hamilton Rating Scale for Depression; MD: Mean difference; MMSE: Mini-Mental State Examination; RR: Risk ratio.  **Explanations:** a. Most risk of bias domains with unclear risk; b. Less than 400 participants; c. 95% CI includes probably relevant and irrelevant effects; d. Does not match optimal information size (OIS) criterion; e. Treatment tolerability assessed by proxy measure f. 95% CI includes probably relevant benefit and harm. | | | | | | | | | | |

Fluvoxamine compared with yokukansan in depressive older patients with AD: GRADE evidence profile.

| **Certainty assessment** | | | | | | **№ of patients** | | **Effect** | | **Certainty** |
| --- | --- | --- | --- | --- | --- | --- | --- | --- | --- | --- |
| **№ of studies** | **Study design** | **Risk of Bias** | **Inconsistency** | **Indirectness** | **Imprecision** | **Fluvoxamine** | **Yokukansan** | **Relative**  **[95% CI]** | **Absolute**  **[95% CI]** |  |
| *Functional status (assessed with: FIM)* | | | | | | | | | | |
| 1 | RCT | Serious ^a^ | Not serious | Not serious | Very serious ^b,c^ | 25 | 26 | - | MD 7.71 pt.  lower  [-24.33, 8.91] | ⨁◯◯◯  VERY LOW |
| *Cognitive function (assessed with: MMSE)* | | | | | | | | | | |
| 1 | RCT | Serious ^a^ | Not serious | Not serious | Very serious ^b,c^ | 25 | 26 | - | MD 0.63 pt.  lower  [-3.43, 2.17] | ⨁◯◯◯  VERY LOW |
| *BPSD (assessed with: NPI-NH)* | | | | | | | | | | |
| 1 | RCT | Serious ^a^ | Not serious | Not serious | Very serious ^b,d^ | 25 | 26 | - | MD 2.16 pt.  lower  [-9.44, 5.12] | ⨁◯◯◯  VERY LOW |
| *Adverse events* | | | | | | | | | | |
| 1 | RCT | Serious ^a^ | Not serious | Not serious | Very serious ^c,e^ | 21/27  (77.8%) | 19/27  (70.4%) | RR 1.11  [0.80, 1.52] | 77 more per  1.000 (from  141 fewer to  366 more) | ⨁◯◯◯  VERY LOW |
| Treatment tolerability (assessed by proxy with total numbers of dropouts) | | | | | | | | | | |
| 1 | RCT | Serious ^a^ | Not serious | Serious | Very serious ^d,e^ | 2/28 (7.1%) | 2/27 (7.4%) | RR 0.96  [0.15, 6.37] | 3 fewer per  1.000 (from 63 fewer to 398 more) | ⨁◯◯◯  VERY LOW |
| *Death, quality of life:* not reported / not assessed. | | | | | | | | | | |
| BPSD: Behavioural and psychological symptoms of dementia; CI: Confidence interval; FIM: Functional Independence Measure; MD: Mean difference; MMSE: Mini-Mental State Examination; NPI-NH: Neuropsychiatric Inventory – Nursing Home Version; RR: Risk ratio.  **Explanations:** a. Most risk of bias domains with unclear risk; b. Less than 400 participants; c. 95% CI includes probably clinically relevant and irrelevant effects; d. 95% CI includes probably clinically relevant benefit and harm; e. Does not match optimal information size (OIS) criterion; f. Treatment tolerability assessed by proxy measure. | | | | | | | | | | |
