# Supplementary material for: Efficacy and safety of pharmacotherapy for Alzheimer’s disease and for behavioural and psychological symptoms of dementia in older patients with moderate and severe functional impairments: a systematic review of controlled trials
Source: Alzheimers Res Ther. 2021 Jul 16;13:131. doi: 10.1186/s13195-021-00867-8 (PMC8285815; doi:10.1186/s13195-021-00867-8)
Supplement: Supplementary file 5 — Additional file 5. [file 13195_2021_867_MOESM5_ESM.docx]

Additional file 5

Anticonvulsants compared with placebo in older patients with AD and agitation: GRADE evidence profile.

| **Certainty assessment** | | | | | | **№ of patients** | | **Effect** | | **Certainty** |
| --- | --- | --- | --- | --- | --- | --- | --- | --- | --- | --- |
| **№ of studies** | **Study design** | **Risk of Bias** | **Inconsistency** | **Indirectness** | **Imprecision** | **Anticonvulsants** | **Placebo** | **Relative**  **[95% CI]** | **Absolute**  **[95% CI]** |  |
| *Functional status (assessed with: PSMS)* | | | | | | | | | | |
| 3 | RCT | Serious ^a^ | Not serious | Not serious | Very serious ^b,c^ | 63 | 64 | - | MD 0.44 pt. higher  [-0.33, 1.22] | ⨁◯◯◯  VERY LOW |
| *Cognitive function (assessed with: MMSE)* | | | | | | | | | | |
| 3 | RCT | Serious ^a^ | Not serious | Not serious | Serious ^b^ | 63 | 64 | - | MD 0.02  pt. lower  [-1.44, 1.47] | ⨁⨁◯◯  LOW |
| *BPSD (assessed with: BPRS)* | | | | | | | | | | |
| 3 | RCT | Serious ^a^ | Not serious | Not serious | Very serious ^b,c^ | 63 | 64 | - | MD 3.02  pt. lower  [-7.62, 1.57] | ⨁◯◯◯  VERY LOW |
| *Adverse events* | | | | | | | | | | |
| 3 | RCT | Serious ^a^ | Not serious | Not serious | Very serious ^c,d^ | 39/64  (60.9%) | 24/64  (37.5%) | RR 1.49  [0.76, 2.93] | 184 more per 1.000  (from 90 fewer to 724  more) | ⨁◯◯◯  VERY LOW |
| *Treatment tolerability (assessed by proxy with total numbers of dropouts)* | | | | | | | | | | |
| 3 | RCT | Serious ^a^ | Not serious | Serious ^e^ | Very serious ^d,f^ | 7/63 (11.1%) | 8/64 (12.5%) | RR 0.94  [0.36, 2.47] | 8 fewer per  1.000 (from 80 fewer to  184 more) | ⨁◯◯◯  VERY LOW |
| *Death, quality of life:* not reported / not assessed. | | | | | | | | | | |
| BPRS: Brief Psychiatric Rating Scale; BPSD: Behavioural and psychological symptoms of dementia; CI: Confidence interval; MD: Mean difference; MMSE: Mini-Mental State Examination; PSMS: Physical Self-Maintenance Scale; RR: Risk ratio.  **Explanations:** a. Most risk of bias domains with unclear risk; b. Less than 400 participants; c. 95% CI includes probably relevant and irrelevant effects; d. Does not match optimal information size (OIS) criterion; e. Treatment tolerability assessed by proxy measure; f. 95% CI includes probably relevant benefit and harm. | | | | | | | | | | |
