# Supplementary material for: Efficacy and safety of pharmacotherapy for Alzheimer’s disease and for behavioural and psychological symptoms of dementia in older patients with moderate and severe functional impairments: a systematic review of controlled trials
Source: Alzheimers Res Ther. 2021 Jul 16;13:131. doi: 10.1186/s13195-021-00867-8 (PMC8285815; doi:10.1186/s13195-021-00867-8)
Supplement: Supplementary file 6 — Additional file 6. [file 13195_2021_867_MOESM6_ESM.docx]

*Additional file 6*

*Haloperidol compared with placebo in older patients with AD and psychotic symptoms: GRADE evidence profile.*

| **Certainty assessment** | | | | | | **№ of patients** | | **Effect** | | **Certainty** |
| --- | --- | --- | --- | --- | --- | --- | --- | --- | --- | --- |
| **№ of studies** | **Study design** | **Risk of Bias** | **Inconsistency** | **Indirectness** | **Imprecision** | **Antidepressants** | **Placebo** | **Relative**  **[95% CI]** | **Absolute**  **[95% CI]** |  |
| *Functional status (assessed with: PSMS)* | | | | | | | | | | |
| 1 | RCT | Serious ^a^ | Not serious | Not serious | Very serious ^b,c^ | 85 | 94 | - | MD 1.12  pt. higher  [0.33, 1.91] | ⨁◯◯◯  VERY LOW |
| *Cognitive function (assessed with: MMSE)* | | | | | | | | | | |
| 1 | RCT | Serious ^a^ | Not serious | Not serious | Serious ^b^ | 63 | 72 | - | MD 0.16 pt. lower  [-1.63, 1.31] | ⨁⨁◯◯  LOW |
| *BPSD (assessed with: NPI-NH)* | | | | | | | | | | |
| 1 | RCT | Serious ^a^ | Not serious | Not serious | Serious ^b^ | 86 | 94 | - | MD 1.82 pt. lower  [-3.51, -0.13] | ⨁⨁◯◯  LOW |
| *Extrapyramidal symptoms (assessed with: SAS)* | | | | | | | | | | |
| 1 | RCT | Serious ^a^ | Not serious | Not serious | Serious ^b^ | 75 | 83 | - | MD 2.72 pt. higher  [1.36, 4.08] | ⨁⨁◯◯  LOW |
| *Death* | | | | | | | | | | |
| 1 | RCT | Serious ^a^ | Not serious | Not serious | Very serious ^d,e^ | 7/94 (7.4%) | 4/98 (4.1%) | RR 1.82  [0.55, 6.03] | 33 more  per 1.000  (from 18 fewer to  205 more) | ⨁◯◯◯  VERY LOW |
| Treatment tolerability (assessed by proxy with total numbers of dropouts) | | | | | | | | | | |
| 1 | RCT | Serious ^a^ | Not serious | Serious ^f^ | Very serious ^c,e^ | 39/94  (41.5%) | 36/99  (36.4%) | RR 1.14  [0.80, 1.63] | 51 more  per 1.000  (from 73  fewer to  229 more) | ⨁◯◯◯  VERY LOW |
| BPSD: Behavioural and psychological symptoms of dementia; CI: Confidence interval; MD: Mean difference; MMSE: Mini-Mental State Examination; NPI-NH: Neuropsychiatric Inventory – Nursing Home Version; PSMS: Physical Self-Maintenance Scale; RR: Risk ratio; SAS: Simpson-Angus-Scale.  **Explanations:** a. Most risk of bias domains with unclear risk; b. Less than 400 participants; c. 95% CI includes probably relevant and irrelevant clinical effects; d. 95% CI includes probably relevant benefit and harm; e. Does not match optimal information size (OIS) criterion; f. Treatment tolerability assessed by proxy measure. | | | | | | | | | | |

*Quetiapine compared with placebo in older patients with AD and psychotic symptoms: GRADE evidence profile.*

| **Certainty assessment** | | | | | | **№ of patients** | | **Effect** | | **Certainty** |
| --- | --- | --- | --- | --- | --- | --- | --- | --- | --- | --- |
| **№ of studies** | **Study design** | **Risk of Bias** | **Inconsistency** | **Indirectness** | **Imprecision** | **Antidepressants** | **Placebo** | **Relative**  **[95% CI]** | **Absolute**  **[95% CI]** |  |
| *Functional status (assessed with: PSMS)* | | | | | | | | | | |
| 1 | RCT | Serious ^a^ | Not serious | Not serious | Very Serious ^b,c^ | 86 | 94 | - | MD 0.48  pt. lower  [-1.13, 0.37] | ⨁◯◯◯  VERY LOW |
| *Cognitive function (assessed with: MMSE)* | | | | | | | | | | |
| 1 | RCT | Serious ^a^ | Not serious | Not serious | Serious ^b^ | 69 | 72 | - | MD 0.68  pt. lower  [-1.92, 0.56] | ⨁⨁◯◯  LOW |
| *BPSD (assessed with: NPI-NH)* | | | | | | | | | | |
| 1 | RCT | Serious ^a^ | Not serious | Not serious | Serious ^b^ | 86 | 94 | - | MD 0.03  pt. lower  [-1.79, 1.73] | ⨁⨁◯◯  LOW |
| *Extrapyramidal symptoms (assessed with: SAS)* | | | | | | | | | | |
| 1 | RCT | Serious ^a^ | Not serious | Not serious | Very Serious ^b,d^ | 73 | 83 | - | MD 0 pt.  [-1.14, 1.14] | ⨁◯◯◯  VERY LOW |
| *Death* | | | | | | | | | | |
| 1 | RCT | Serious ^a^ | Not serious | Not serious | Very Serious ^d,e^ | 2/91 (2.2%) | 4/98 (4.1%) | RR 0.54  [0.10, 2.87] | 19 fewer  per 1.000  (from 37 fewer to 76 more) | ⨁◯◯◯  VERY LOW |
| Treatment tolerability (assessed by proxy with total numbers of dropouts) | | | | | | | | | | |
| 1 | RCT | Serious ^a^ | Not serious | Serious ^f^ | Very Serious ^d,e^ | 29/91  (31.9%) | 36/99  (36.4%) | RR 0.88  [0.59, 1.30] | 44 fewer  per 1.000  (from 149  fewer to 109  more) | ⨁◯◯◯  VERY LOW |
| BPSD: Behavioural and psychological symptoms of dementia; CI: Confidence interval; MD: Mean difference; MMSE: Mini-Mental State Examination; NPI-NH: Neuropsychiatric Inventory – Nursing Home Version; PSMS: Physical Self-Maintenance Scale; RR: Risk ratio; SAS: Simpson-Angus-Scale.  **Explanations:** a. Most risk of bias domains with unclear risk; b. Less than 400 participants; c. 95% CI includes probably relevant and irrelevant clinical effects; d. 95% CI includes probably relevant benefit and harm; e. Does not match optimal information size (OIS) criterion; f. Treatment tolerability assessed by proxy measure. | | | | | | | | | | |

*Risperidone compared with yokukansan in older patients with AD and BPSD: GRADE evidence profile.*

| **Certainty assessment** | | | | | | **№ of patients** | | **Effect** | | **Certainty** |
| --- | --- | --- | --- | --- | --- | --- | --- | --- | --- | --- |
| **№ of studies** | **Study design** | **Risk of Bias** | **Inconsistency** | **Indirectness** | **Imprecision** | **Antidepressants** | **Placebo** | **Relative**  **[95% CI]** | **Absolute**  **[95% CI]** |  |
| *Functional status (assessed with: FIM)* | | | | | | | | | | |
| 1 | RCT | Serious ^a^ | Not serious | Not serious | Very serious ^b,c^ | 25 | 26 | - | MD 1.15  pt. lower  [-17.08, 14.78] | ⨁◯◯◯  VERY LOW |
| *Cognitive function (assessed with: MMSE)* | | | | | | | | | | |
| 1 | RCT | Serious ^a^ | Not serious | Not serious | Very serious ^b,d^ | 25 | 26 | - | MD 0.47 pt.  lower  [-3.04, 2.1] | ⨁◯◯◯  VERY LOW |
| *BPSD (assessed with: NPI-NH)* | | | | | | | | | | |
| 1 | RCT | Serious ^a^ | Not serious | Not serious | Very serious ^b,c^ | 25 | 26 | - | MD 2.72  pt. higher  [-3.34, 8.87] | ⨁◯◯◯  VERY LOW |
| *Extrapyramidal symptoms (assessed with: DIEPSS)* | | | | | | | | | | |
| 1 | RCT | Serious ^a^ | Not serious | Not serious | Serious ^b^ | 25 | 26 | - | MD 0.87 pt. higher  [0.43, 1.31] | ⨁⨁◯◯  LOW |
| *Death* | | | | | | | | | | |
| 1 | RCT | Serious ^a^ | Not serious | Not serious | Very serious ^c,e^ | 1/27 (3.7%) | 0/27 (0.0%) | RR 3.00  [0.13, 70.53] | 0 fewer per 1.000.000  (from 0 fewer to 0 fewer) | ⨁◯◯◯  VERY LOW |
| Treatment tolerability (assessed by proxy with total numbers of dropouts) | | | | | | | | | | |
| 1 | RCT | Serious ^a^ | Not serious | Serious ^f^ | Very serious ^c,e^ | 2/28 (7.1%) | 1/27 (3.7%) | RR 1.93  [0.19, 20.05] | 34 more  per 1.000  (from 30 fewer to 706 more) | ⨁◯◯◯  VERY LOW |
| BPSD: Behavioural and psychological symptoms of dementia; CI: Confidence interval; DIEPSS: Drug-Induced Extrapyramidal Symptom Scale; FIM: Functional Independence Measure; MD: Mean difference; MMSE: Mini-Mental State Examination; RR: Risk ratio; SAS: Simpson-Angus-Scale.  **Explanations:** a. Most risk of bias domains with unclear risk; b. Less than 400 participants; c. 95% CI includes probably relevant benefit and harm; d. 95% CI includes probably relevant and irrelevant clinical effects; e. Does not match optimal information size (OIS) criterion; f. Treatment tolerability assessed by proxy measure. | | | | | | | | | | |
