# Supplementary material for: Efficacy and safety of pharmacotherapy for Alzheimer’s disease and for behavioural and psychological symptoms of dementia in older patients with moderate and severe functional impairments: a systematic review of controlled trials
Source: Alzheimers Res Ther. 2021 Jul 16;13:131. doi: 10.1186/s13195-021-00867-8 (PMC8285815; doi:10.1186/s13195-021-00867-8)
Supplement: Supplementary file 7 — Additional file 7. [file 13195_2021_867_MOESM7_ESM.docx]

| Database | Search strategy |
| --- | --- |
| **Embase** | 1. random*.tw. 2. clinical trial*.mp. 3. exp health care quality/ 4. 1 or 2 or 3 5. Elder*.tw,kw. 6. (community adj1 dwelling).tw,kw. 7. Geriatric.tw,kw. 8. "mini-mental state".tw,kw. 9. alzheimer*.tw,kw. 10. mmse.tw,kw. 11. caregiver.tw,kw. 12. falls.tw,kw. 13. adl.tw,kw. 14. frail*.tw,kw. 15. Gds.tw,kw. 16. Ag?ing.tw,kw. 17. Mci.tw,kw. 18. dement*.tw,kw. 19. (psychogeriatric* or psycho-geriatric*).tw,kw. 20. "cognitive impairment".tw,kw. 21. „postmenopausal women“.tw,kw. 22. comorbid*.tw,kw. 23. nursing home/ 24. geriatric assessment/ 25. frail elderly/ 26. Alzheimer disease/ep [Epidemiology] 27. Exp cognitive defect/di [Diagnosis] 28. Exp cogntive defect/ep [Epidemiology] 29. Home for the aged/ 30. disability.tw,kw. 31. „functional decine“.tw,kw. 32. (gerontopsychiatry or geronto-psychiatry).tw,kw. 33. „activities of daily living“.tw,kw. 34. immobility.tw,kw. 35. Daily life activity/ 36. exp dementia/ 37. immobilization.tw,kw. 38. disabled person/ 39. or/5-38 40. MCI.tw,kw. 41. cognit* impair*.tw,kw. 42. Alzheimer Disease Assessment Scale/ 43. Dementia/ or Alzheimer disease/ or diffuse neurofibrillary tangles with calcification/ or mental deterioration/ or „mixed depressio and dementia“/ or exp senile dementia/ or tauopathy/ 44. exp cognitive defect/ 45. dement.tw,kw. 46. alzheimer*.tw,kw. 47. („organic brain disease“ or „organic brain syndrome“).tw,kw. 48. "benign senescent forgetfulness".tw,kw. 49. neurodegenerat*.tw,kw. 50. (cerebral* adj2 insufficient*).tw,kw. 51. exp memory disorder/ 52. exp cognition/ 53. delusion.tw,kw. 54. irritability.tw,kw. 55. hallucination*.tw,kw. 56. depression*.tw,kw. 57. dysphoria.tw,kw. 58. agitation.tw,kw. 59. deliri*.tw,kw. 60. agression.tw,kw. 61. „BPSD“.tw,kw. 62. „Behavioral and Psychological Symptoms of Dementia“.tw,kw. 63. (behavio* adj3 psycholog* adj3 symptom* adj3 dement*).mp. 64. (neuropsychiatric or neuro-psychiatric).tw,kw. 65. (neurobehavioral or neur-behavioral).tw,kw. 66. or/53-65 67. 42 or 43 or 46 68. 66 and 67 69. or/40-52 70. 68 or 69 71. exp cholinesterase inhibitor/ 72. (acetylcholinesterase inhibitor* or cholinesterase inhibitor* or anticholinesteras* or anti-cholinesteras*).tw,kw. 73. donepezil*.tw,kw. 74. aricept*.tw,kw. 75. donepezil/ 76. galantamine/ 77. galanthamin*.tw,kw. 78. galantamin*.tw,kw. 79. reminyl*.tw,kw. 80. nivalin*.tw,kw. 81. razadyne*.tw,kw. 82. rivastigmine/ 83. rivastigmin*.tw,kw. 84. exelon*.tw,kw. 85. tacrine/ 86. tacrin*.tw,kw. 87. cognex*.tw,kw. 88. (anti-dementia drug* or antidementia drug* or memory drug*).tw,kw. 89. ((anti-alzheimer* or antialzheimer) adj2 drug*).tw,kw. 90. memantine/ 91. memantin*.tw,kw. 92. axura*.tw,kw. 93. ebixa.tw.kw. 94. namenda*.tw,kw. 95. Ginkgo biloba/ 96. ginkgo.tw,kw. 97. „EGb 761“.tw,kw. 98. aripiprazole/ 99. aripiprazol*.tw,kw. 100. haloperidol/ 101. haloperidol*.tw,kw 102. quetiapine/ 103. quetiapin*.tw,kw. 104. risperidone/ 105. risperidon*.tw,kw. 106. carbamazepine/ 107. carbamazepin*.tw,kw. 108. citalopram/ 109. citalopram*.tw,kw. 110. paroxetine/ 111. paroxetin*.tw,kw. 112. sertraline/ 113. sertralin*.tw,kw. 114. mirtazapine/ 115. mirtazapin*.tw,kw. 116. pipamperone/ 117. pipamperon*.tw,kw. 118. melperone/ 119. melperon*.tw,kw. 120. drug therap*.tw,kw. 121. (pharmacotherap* or pharmaco-thrap*).tw,kw. 122. drugs.tw,kw. 123. Medication.tw,kw. 124. drug therapy/ 125. or/120-124 126. or/71-119 127. (70 and 125) or 126 128. 4 and 39 and 127 129. limit 128 to human 130. limit 129 to exclude medline journals 131. limit 130 to yr=“1992 –Current“ |
| **Ovid MEDLINE Epub Ahead of Print, In-Process & Other Non-Indexed Citations, Ovid MEDLINE Daily and Ovid MEDLINE** | 1. randomized controlled trial.pt. 2. controlled clinical trial.pt. 3. randomi#ed.ab. 4. placebo.ab. 5. drug therapy.fs. 6. randomly.ab. 7. groups.ab. 8. trial.ab. 9. exp animals/ not humans.sh. 10. 1 or 2 or 3 or 4 or 5 or 6 or 7 or 8 11. 10 not 9 12. elder*.ti,ab,kf. 13. (community adj1 dwelling).ti,ab,kf. 14. geriatric.ti,ab,kf. 15. „mini-mental state“.ti,ab,kf. 16. alzheimer*.ti,ab,kf. 17. mmse.ti,ab,kf. 18. caregiver*.ti,ab,kf. 19. falls.ti,ab,kf. 20. adl.ti,ab,kf. 21. frail*.ti,ab,kf. 22. Gds.ti,ab,kf. 23. Ag?ing.ti,ab,kf. 24. Mci.ti,ab,kf. 25. Dement*.ti,ab,kf. 26. (psycho-geriatric* or psychogeriatric*).ti,ab,kf. 27. „cognitive impairment“.ti,ab,kf. 28. "postmenopausal women".ti,ab,kf. 29. comorbid*.ti,ab,kf. 30. exp Nursing Homes/ 31. Geriatric assessement/ 32. Frail Elderly/ 33. exp Cognition Disorders/di 34. exp Cognition disorders/ep 35. Homes for the Aged/ 36. disability.ti,ab,kf. 37. „functional decline“.ti,ab,kf. 38. (gerontopsychiatry or geronto-psychiatry).ti,ab,kf. 39. „activities of daily living“.ti,ab,kf. 40. immobility.ti,ab,kf. 41. exp „Activities of daily living“/ 42. exp dementia/ 43. immobilization.ti,ab,kf. 44. disabled persons/ or persons with hearing impairments/ or visually impaired persons/ 45. or/12-44 46. MCI.ti,ab,kf. 47. Cognit* impair*.ti,ab,kf. 48. alzheimer disease/ 49. Cognitive Dysfunction/ 50. Cognition disorders/ 51. Cognition/ 52. Dementia/ 53. Dement*.ti,ab,kf. 54. Alzheimer*.ti,ab,kf. 55. („organic brain disease“ or „organic brain syndrome“).ti,ab,kf. 56. "benign senescent forgetfulness".ti,ab,kf. 57. Neurodegenerat*.ti,ab,kf. 58. (cerebral* adj2 insufficient*).ti,ab,kf. 59. Memory Disorders/ 60. Amnesia/ 61. Depression*.ti,ab,kf. 62. Delusion*.ti,ab,kf. 63. Dysphoria.ti,ab,kf. 64. Agitation.ti,ab,kf. 65. Hallucination.ti,ab,kf. 66. Irritability.ti,ab,kf. 67. Aggression.ti,ab,kf. 68. „BPSD“.ti,ab,kf. 69. (behavio* adj3 psycholog* adj3 symptom* adj3 dement*).mp. 70. "Behavioral and Psychological Symptoms of Dementia".ti,ab,kf. 71. Neuropsychiatric.ti,ab,kf. 72. neuro-psychiatric.ti,ab,kf. 73. Neurobehavioral.ti,ab,kf. 74. neuro-behavioral.ti,ab,kf. 75. Deliri*.ti,ab,kf. 76. or/61-75 77. 48 or 52 or 54 78. 76 and 77 79. or/46-60 80. 78 or 79 81. Cholinesterase inhibitors/ 82. (acetylcholinesterase inhibitor* or cholinesterase inhibitor* or anti-cholinesteras* or anticholinesteras*).ti,ab,kf,nm. 83. donepezil*.ti,ab,kf,nm. 84. aricept*.ti,ab,kf,nm. 85. Galantamine/ 86. galanthamin*.ti,ab,kf,nm. 87. Galatamin*.ti,ab,kf,nm. 88. Reminyl*.ti,ab,kf,nm. 89. Nivalin*.ti,ab,kf,nm. 90. Razadyne*.ti,ab,kf,nm. 91. Rivastigmin*.ti,ab,kf,nm. 92. exelon*.ti,ab,kf,nm. 93. Tacrine/ 94. tacrin*.ti,ab,kf,nm. 95. Cognex*.ti,ab,kf,nm. 96. (anti-dementia drug* or antidementia drug* or memory drug).ti,ab,kf,nm. 97. ((anti-alzheimer* or antialzheimer) adj2 drug*).ti,ab,kf,nm. 98. Memantine/ 99. Rivastigmine/ 100. Galanthamine/ 101. Memantin*.ti,ab,kf,nm. 102. namenda*.ti,ab,kf,nm. 103. Axura.ti,ab,kf,nm. 104. ebixa*.ti,ab,kf,nm. 105. Ginkgo.ti,ab,kf,nm. 106. "EGb 761".ti,ab,kf,nm. 107. Ginkgo biloba/ 108. aripiprazole/ 109. quetiapin fumarate/ 110. risperidone/ 111. haloperidol/ 112. aripiprazol*.ti,ab,kf,nm. 113. Haloperidol*.ti,ab,kf,nm. 114. quetiapin*.ti,ab,kf,nm. 115. Risperidon*.ti,ab,kf,nm. 116. carbamazepine/ 117. carbamazepin*.ti,ab,kf,nm. 118. citalopram/ 119. Sertraline/ 120. Paroxetine/ 121. Sertralin*.ti,ab,kf,nm. 122. mirtazapin*.ti,ab,kf,nm. 123. Citalopram*.ti,ab,kf,nm. 124. paroxetin*.ti,ab,kf,nm. 125. Pipamperon*.ti,ab,kf,nm. 126. melperon*.ti,ab,kf,nm. 127. Drug therap*.ti,ab,kf,fs. 128. (pharmcotherap* or pharmaco-therap*).ti,ab,kw. 129. Drugs.ti,ab,kf. 130. Medication.ti,ab,kf. 131. Drug therapy/ or drug administration routes/ or drug administration schedule/ or drug delivery systems/ or drug dose calculations/ or drug prescription/ or drug therapy, combination/ or drug therapy, computer-assisted/ or inappropriate prescribing/ or medication errors/ or polypharmacy/ or self administration/ or self medication/ 132. or/127-131 133. or/81-126 134. (80 and 132) or 133 135. 11 and 45 and 134 136. Limit 135 to (humans and yr=“1992 –Current“) |
| **Cochrane Central Register of Controlled Trials (CENTRAL)** | 1. MCI 2. cognit* next impair* 3. MeSH descriptor: [Alzheimer disease] this term only 4. MeSH descriptor: [Dementia] this term only 5. MeSH descriptor: [Cognition] this term only 6. MeSH descriptor: [Cognition disorders] this term only 7. MeSH descriptor: [Cognitive dysfunction] this term only 8. MeSH descriptor: [Memory disorders] this term only 9. MeSH descriptor: [Amnesia] this term only 10. Dement* 11. alzheimer* 12. ("organic brain disease" or "organic brain syndrome") 13. "benign senescent forgetfulness" 14. cerebral* near/2 insufficient* 15. neurodegenerat* 16. delusion* 17. hallucination* 18. dysphoria 19. depression 20. irritability 21. agitation 22. aggression 23. BPSD 24. neuropsychiatric or neuro-psychiatric 25. "Behavioral and Psychological Symptoms of Dementia" 26. neurobehavioral or neuro-behavioral 27. deliri* 28. #3 or #4 or #11 29. #16 or #17 or #18 or #19 or #20 or #21 or #22 or #23 or #24 or #25 or #26 or #27 30. #28 and #29 31. #1 or #2 or #3 or #4 or #5 or #6 or #7 or #8 or #9 or #10 or #11 or #12 or #13 or #14 or #15 or #30 32. MeSH descriptor: [Cholinesterase Inhibitors] this term only 33. acetylcholinesterase next inhibitor* 34. cholinesterase next inhibitor* 35. anti-cholinesteras* or anticholinesteras* 36. donepezil* 37. aricept* 38. MeSH descriptor: [Galantamine] this term only 39. galanthamin* 40. galantamin* 41. Reminyl* 42. Nivalin* 43. Razadyne* 44. rivastigmin* 45. MeSH descriptor: [Rivastigmine] this term only 46. exelon* 47. MeSH descriptor: [Tacrine] this term only 48. tacrin* 49. cognex* 50. anti-dementia next drug* or antidementia next drug* 51. memory next drug* 52. anti-alzheimer near/2 drug* or antialzheimer near/2 drug* 53. MeSH descriptor: [Memantine] this term only 54. memantin* 55. namenda* 56. axura* 57. ebixa* 58. ginkgo 59. "EGb 761" 60. MeSH descriptor: [Ginkgo biloba] this term only 61. MeSH descriptor: [Aripiprazole] this term only 62. MeSH descriptor: [Haloperidol] this term only 63. MeSH descriptor: [Quetiapine Fumarate] this term only 64. MeSH descriptor: [Risperidone] this term only 65. aripiprazol* 66. haloperidol* 67. quetiapin* 68. risperidon* 69. MeSH descriptor: [Carbamazepine] this term only 70. carbamazepin* 71. MeSH descriptor: [Citalopram] this term only 72. MeSH descriptor: [Paroxetine] this term only 73. MeSH descriptor: [Sertraline] this term only 74. Sertralin* 75. mirtazapin* 76. paroxetin* 77. melperon* 78. pipamperon* 79. citalopram* 80. #32 or #33 or #34 or #35 or #36 or #37 or #38 or #39 or #40 or #41 or #42 or #43 or #44 or #45 or #46 or #47 or #48 or #49 or #50 or #51 or #52 or #53 or #54 or #55 or #56 or #57 or #58 or #59 or #60 or #61 or #62 or #63 or #64 or #65 or #66 or #67 or #68 or #69 or #70 or #71 or #72 or #73 or #74 or #75 or #76 or #77 or #78 or #79 81. drug next therap* 82. medication 83. drugs 84. MeSH descriptor: [Drug Therapy] this term only 85. MeSH descriptor: [Drug Administration Routes] this term only 86. MeSH descriptor: [Drug Administration Schedules] this term only 87. MeSH descriptor: [Drug Delivery Systems] this term only 88. MeSH descriptor: [Drug Dose Calculations] this term only 89. MeSH descriptor: [Drug Prescriptions] this term only 90. MeSH descriptor: [Drug Therapy, Combination] this term only 91. MeSH descriptor: [Drug Therapy, Computer-Assisted] this term only 92. MeSH descriptor: [Inappropriate Prescribing] this term only 93. MeSH descriptor: [Medication Errors] this term only 94. MeSH descriptor: [Polypharmacy] this term only 95. MeSH descriptor: [Self Administration] this term only 96. MeSH descriptor: [Self Medication] this term only 97. Pharmacotherap* or pharmaco-therap* 98. #81 or #82 or #83 or #84 or #85 or #86 or #87 or #88 or #89 or #90 or #91 or #92 or #93 or #94 or #95 or #96 or #97 99. #31 and #98 100. #99 or #80 101. elder* 102. community near/1 dwelling 103. geriatric 104. "mini-mental state" 105. alzheimer* 106. mmse 107. caregiver* 108. falls 109. Adl 110. Frail* 111. Gds 112. Ag?ing 113. Mci 114. dement* 115. psycho-geriatric* or psychogeriatric* 116. cognitive next impairment 117. postmenopausal next women 118. comorbid* 119. MeSH descriptor: [Geriatric Assessment] this term only 120. MeSH descriptor: [Nursing homes] explode all trees 121. MeSH descriptor: [Frail Elderly] this term only 122. MeSH descriptor: [Cognition disorders] explode all trees with qualifier(s): [Diagnosis – DI] 123. MeSH descriptor: [Cognition disorders] explode all trees with qualifier(s): [Epidemiology - EP] 124. MeSH descriptor: [Homes for the Aged] this term only 125. Disability 126. functional next decline 127. gerontopsychiatry or geronto-psychiatry 128. "activities of daily living" 129. Immobility 130. MeSH descriptor: [Activities of Daily Living] explode all trees 131. MeSH descriptor: [Dementia] explode all trees 132. Immobilization 133. MeSH descriptor: [Disabled Persons] this term only 134. MeSH descriptor: [Persons With Hearing Impairments] this term only 135. MeSH descriptor: [Visually Impaired Persons] this term only 136. #101 or #102 or #103 or #104 or #105 or #106 or #107 or #108 or #109 or #110 or #111 or #112 or #113 or #114 or #115 or #116 or #117 or #118 or #119 or #120 or #121 or #122 or #123 or #124 or #125 or #126 or #127 or #128 or #129 or #130 or #131 or #132 or #133 or #134 or #135 137. #100 and #136 (Publication Year from 1992, in Trials) |
